# Effect of sleep apnoea interventions on multiple health outcomes: an umbrella review of meta-analyses of randomised controlled trials

**DOI:** 10.1016/j.eclinm.2025.103529

**Published:** 2025-10-08

**Authors:** Camille Figard, Raoua Ben Messaoud, Sébastien Baillieul, Marie Joyeux-Faure, Marie Destors, Renaud Tamisier, Charles Khouri, Jean-Louis Pépin

**Affiliations:** a EFCR Laboratory, Thorax and Vessels Division, Grenoble Alpes University Hospital, Grenoble, France; b HP2 Laboratory, Inserm U1300, University Grenoble Alpes, Grenoble, France; c Regional Pharmacovigilance Center & Clinical Pharmacology Unit, Grenoble Alpes University Hospital, University Grenoble Alpes, Grenoble, France

**Keywords:** Obstructive sleep apnoea, Treatment, Meta-analysis, Randomised controlled trials, Efficacy

## Abstract

**Background:**

Obstructive sleep apnoea (OSA) is a prevalent chronic condition that is associated with cardiometabolic and neurocognitive complications. While continuous positive airway pressure (CPAP) remains the first-line therapy, suboptimal adherence limits its effectiveness, highlighting the need to evaluate alternatives such as mandibular advancement devices (MADs), hypoglossal nerve stimulation (HNS), physical activity, different modalities of weight loss management including glucagon-like peptide-1 (GLP-1) agonists and combination therapies.

**Methods:**

We conducted an umbrella review to synthesise high-level evidence from meta-analyses of randomised controlled trials (RCTs) evaluating the efficacy, adherence, and safety of therapies used in patients with OSA. A comprehensive search was performed in PubMed, Embase, Web of Science, and the Cochrane Database of Systematic Reviews covering the period from January 1, 2017, to July 5, 2025. Eligible studies were meta-analyses published in English assessing interventions targeting key OSA outcomes, including changes in apnoea–hypopnoea index (AHI), Epworth Sleepiness Scale (ESS), quality of life (QoL), blood pressure (BP), treatment adherence, and safety. When multiple meta-analyses addressed the same intervention-outcome pair, the one including the highest number of RCTs was retained. Two reviewers independently screened studies and extracted data. Methodological quality was assessed using the AMSTAR 2 tool, and the certainty of evidence was evaluated using the GRADE framework. Meta-analyses published in languages other than English, those focusing on paediatric populations or interventions outside the scope of conventional OSA management, and meta-analyses that did not report any of the pre-specified outcomes/interventions of interest were excluded. The review protocol was registered in PROSPERO (CRD42023420729) and the Open Science Framework (https://osf.io/2jvsx).

**Findings:**

A total of 5571 meta-analyses were identified. Of these, 34 met the inclusion criteria, encompassing 230 RCTs and 36,353 participants (n = 26,058 [72.3%] male). GRADE assessment showed that 12 meta-analyses (35%) had evidence that was of low certainty, 23 (68%) provided moderate-certainty evidence, and only one (3%) provided evidence that was of high certainty. CPAP was the most effective treatment for reducing AHI (mean difference [MD] −30.7 events/h; standardised mean difference [SMD] −1.65, 95% confidence interval [CI] −1.87 to −1.43; low-certainty evidence), followed by GLP-1 receptor agonists (tirzepatide: MD –21.86 events/h; SMD –0.84, 95% CI –1.01 to −0.68; moderate-certainty evidence) and MADs (MD –11.91 events/h; SMD –0.73, 95% CI –14.25 to −9.75; low-certainty evidence). CPAP, wake stimulants, HNS, and myofunctional therapy significantly reduced daytime sleepiness (ESS score SMDs of −0.80 to −0.88; moderate-certainty evidence except for pitolisant and solriamfetol, which were supported by high-certainty evidence). Physical activity led to the greatest improvements in QoL (SMD 1.3, 95% CI 0.58 to 2.02; moderate-certainty evidence), while CPAP also showed modest benefits (SMD 0.16, 95% CI 0.11 to 0.21; critically low-certainty evidence).

**Interpretation:**

This umbrella review identified CPAP as the most effective intervention for reducing AHI and daytime sleepiness in patients with OSA, while physical activity yielded the greatest improvements in quality of life. Data on safety, long-term adherence, and combination therapies remain scarce, underscoring the need for more comparative and longitudinal research to support personalised treatment strategies. Data need to be interpreted in the context of several limitations, including those relating to the meta-analysis inclusion criteria and the quality of data in the meta-analyses themselves.

**Funding:**

None.

**Research in context**

**Evidence before this study**

We searched four major databases (PubMed, Embase, Cochrane Library, Web of Science) and the PROSPERO and OFS for English-language publications between January 1, 2017 and July 5, 2025 to identify any umbrella reviews of treatments for obstructive sleep apnoea (OSA). None were found. The search strategy combined terms for OSA (“obstructive sleep apnoea” OR “OSA” OR “sleep apnoea” [MeSH]) with terms for treatment (“treatment” OR “therapy”) and methodological filters (“meta-analysis” OR “systematic review” OR “systematic overview” OR “meta analy” OR “metaanaly∗” OR “systematic review/overview”∗). Intervention-specific keywords included: stimulants (solriamfetol, pitolisant, modafinil), mandibular advancement devices (MADs) and oral appliances, continuous positive airway pressure (CPAP), weight loss and diet, physical activity and exercise, bariatric surgery, glucagon-like peptide-1 (GLP-1) receptor agonists (liraglutide, tirzepatide), and non-pharmacological approaches such as myofunctional therapy, oropharyngeal therapy, respiratory muscle training, and orofacial therapy. Boolean operators were applied to combine these terms systematically. To date, only individual meta-analyses on specific interventions such as continuous positive airway pressure (CPAP), mandibular advancement devices (MADs), hypoglossal nerve stimulation (HNS), physical activity, and pharmacological therapies including glucagon-like peptide-1 (GLP-1) receptor agonists have been published. Although these reviews provide valuable insights, their findings remain fragmented, with variable methodological quality and certainty of evidence. Since our initial search, one broader synthesis, a network meta-analysis published in 2025, has become available, but it compared only selected interventions and did not provide an umbrella-level synthesis. This highlights the absence of an overarching review integrating the totality of available evidence regarding treatments used in patients with OSA, and thus the need for the present umbrella meta-analysis.

**Added value of this study**

We systematically evaluated and compared meta-analyses covering 230 randomised controlled trials on the efficacy, adherence, and safety of a wide range of interventions for OSA. We confirmed the superiority of CPAP in reducing the apnoea–hypopnoea index (AHI) and daytime sleepiness and showed that structured physical activity resulted in the greatest improvements in quality of life (QoL). Evidence for emerging therapies, such as GLP-1 receptor agonists and HNS, was promising but limited by the availability of small numbers of trials that had short follow-up durations. By critically appraising methodological quality and integrating findings across domains, this study provides the first high-level comparative overview of both established and novel treatments used in patients with OSA.

**Implications of all the available evidence**

It appears possible that optimal approaches to OSA management may come from considering alternative and combination treatments that are tailored to the risk profile of each individual, moving beyond a solely CPAP-focused, “one size fits all” CPAP strategy. Adjunctive interventions such as physical activity and novel pharmacotherapies show promise, although the certainty of supporting evidence remains variable, and in some cases limited. Therefore, data on the suitability and effectiveness of alternative and additional options for the management of OSA should be interpreted with caution. As a result, these should be considered as complementary, rather than definitive, options. Future research should focus on strengthening the quality of evidence, particularly regarding the durability of treatment effects, long-term safety, and the potential synergistic benefits of combination therapies. While the current umbrella review provides a comparative synthesis of evidence across interventions, the implications of its findings for clinical practice and policy should be viewed in the context of the heterogeneity, risk of bias, and variable certainty of evidence from the included meta-analyses.

## Introduction

Obstructive sleep apnoea (OSA) is a highly prevalent chronic disease affecting nearly one billion people aged 30–69 years worldwide.^1^ OSA is characterised by the repetitive occurrence of partial (hypopnoeas) or complete (apnoeas) pharyngeal collapses during sleep.^1^ In addition to causing symptoms such as sleepiness, fatigue and impaired mood,^2^ untreated OSA is associated with serious health consequences including cardiometabolic diseases, neurocognitive dysfunction, and early mortality.^3^, ^4^, ^5^ The substantial individual, societal and health systems burden of OSA mean that it is essential to provide effective therapies to mitigate the condition's overall impact on both individual quality of life (QoL) and health-related costs.^6^

According to current guidelines,^7^ continuous positive airway pressure (CPAP) represents the first-line therapy for moderate to severe OSA. The efficacy of CPAP is well established in terms of improvement in symptoms and quality of life. However, the real-world effectiveness of CPAP is reduced by challenges in maintaining long-term adherence and high rates of therapy termination.^8^^,^^9^ Recent approaches to improving the treatment of OSA include the identification of specific disease phenotypes and endotypes,^10^^,^^11^ which have highlighted the importance of holistic and personalised approaches to OSA management. A key feature of these approaches is the incorporation of alternatives to CPAP therapy that may be more effective in some specific OSA subtypes due to greater patient acceptance.^12^

The most widely used alternative interventions include mandibular advancement devices (MADs),^13^ positional therapy^14^^,^^15^ oral and maxillofacial surgery,^16^ oropharyngeal myofunctional therapy (OMT),^17^^,^^18^ different approaches to weight loss,^19^ physical activity,^20^^,^^21^ hypoglossal nerve stimulation (HNS)^22^ and, more recently, wakefulness-promoting agents^23^ and glucagon-like peptide-1 (GLP-1) agonists.^24^, ^25^, ^26^ Furthermore, there is growing awareness of the need to combine intervention modalities.^27^

The effectiveness of treatment for OSA is primarily evaluated by assessment of sleep study data, including parameters such as the apnoea–hypopnoea index (AHI), which reflects the number of abnormal respiratory events during sleep. Other important study endpoints include daytime sleepiness (evaluated using the Epworth Sleepiness Scale [ESS]), quality of life (QoL), and intermediate and objective cardiovascular outcomes such as blood pressure (BP).

Given the large number of randomised controlled trials (RCTs), systematic reviews and meta-analyses available in the field of OSA treatment, there is a need for a summary of the best evidence for currently available interventions that can be used to guide clinician decision-making about the most appropriate approach for each individual patient. A network meta-analysis has recently been published,^28^ that focused on selected treatments and did not provide a comprehensive, umbrella-level synthesis. An effective way to address this gap is through an umbrella review (also known as overview of systematic reviews), which offers a higher-level evidence synthesis while also highlighting uncertainties, potential biases, and knowledge gaps.^29^

To assimilate the vast amount of research on treatments for OSA, this umbrella review of RCT data comprehensively evaluated the impact of the main therapies and interventions used in patients with OSA on efficacy outcomes, adherence and safety.

## Methods

### Search strategy and selection criteria

The study protocol was prospectively registered in both PROSPERO (CRD42023420729) and the Open Science Framework (https://osf.io/2jvsx). We reported the results according to the preferred reporting items for overviews of reviews (PRIOR) statement.^30^ No ethical approval was required for this meta-analysis because it involved the collection and synthesis of data from previously conducted clinical trials where informed consent had already been obtained by the original investigators. Our review was conducted in accordance with the PRISMA (Preferred Reporting Items for Systematic Reviews and Meta-Analyses) guidelines. The search strategy reporting also followed the PRISMA-S extension to ensure transparency and reproducibility.

We conducted a comprehensive systematic search of Medline (via PubMed), Embase, Web of science and Cochrane Database of Systematic Reviews for the period January 1, 2017, to July 5, 2025, to identify recent systematic reviews and meta-analyses of RCTs assessing the efficacy and safety of OSA interventions. In addition, we manually screened the reference lists of all included articles to identify any further relevant studies. When selected studies appeared to be crucial but lacked essential information, we also contacted the corresponding authors by email or via other channel to request clarification or additional. Full details of the search strategy are provided in Supplementary Appendix Table A.

Meta-analyses of RCTs published in English since 2017 were selected based on the following PICO (Population, Intervention, Comparator, Outcome) strategy:

(1) Population: adults with OSA;
(2) Interventions: conventional OSA treatments (CPAP, MAD, oral and maxillofacial surgery), weight loss strategies (bariatric surgery, diet), GLP-1 agonists, physical activity, positional therapy, HNS, OMT and wakefulness-promoting agents (pitolisant, solriamfetol, modafinil);
(3) Comparators: inactive control (sham CPAP, placebo, no intervention), or any of the conventional interventions listed above;
(4) Outcomes: efficacy (OSA severity [based on the AHI], symptoms [daytime sleepiness based on the ESS], QoL, BP, MACE), safety (adverse events) and adherence.

Full details of each of these outcomes and measurements are provided in Supplementary Appendix Table B.

Studies were excluded if they did not align with one of the components of the defined PICO framework or lacked quantitative analysis. A list of eligible meta-analyses that were subsequently excluded based on either an insufficient number of RCTs for each intervention-outcome combination or AMSTAR 2 assessment is provided in Supplementary Appendix Table C.

The selection process was conducted in multiple systematic steps to ensure transparency, reproducibility, and the robustness of our umbrella review. Two authors (CF and RBM) independently screened all titles and abstracts using the Rayyan platform (https://www.rayyan.ai/), which allows for blinded and collaborative manual screening. No automated or machine-assisted methods were used; all inclusion and exclusion decisions were based on manual assessment. While a formal librarian review was not conducted, we employed a comprehensive multi-database search strategy and sought input from domain experts to ensure thorough coverage of the literature.

All studies identified as potentially eligible underwent manual full-text review before final inclusion. The reference lists of relevant studies were also screened manually. Any discrepancies were resolved by consensus with two other authors (CK and JLP). We classified all identified meta-analyses by intervention-outcome combinations. At this stage, we included only quantitative meta-analyses of RCTs that reported results on predefined intervention-outcome combinations relevant to the treatment of OSA. To avoid duplication and overlapping data, we classified all eligible meta-analyses according to specific intervention-outcome pairs. When multiple meta-analyses addressed the same combination, we retained the one that included the largest number of RCTs to ensure comprehensive coverage of the available evidence. In instances where the number of RCTs was similar or the included primary studies overlapped substantially, the meta-analysis with the highest methodological quality, as assessed using the AMSTAR 2 tool, was selected.

For this umbrella review, we included only outcomes that were assessed in studies involving at least two different active interventions, even if each was separately compared with an inactive control (i.e. the two active treatments were not directly compared in the source meta-analysis).

### Data analysis

Two authors (CF and RBM) independently extracted the data; discrepancies were settled by a third author (CK) when needed. The following information was collected from the meta-analyses and original studies: first author, journal and year of publication, number of included RCTs, outcome(s) of interest, type of interventions and controls. We also extracted all effect estimates with 95% confidence interval (CI) values for original studies pooled in the meta-analyses and the pooled effect size metric (hazard ratio [HR], odds ratio [OR], risk ratio [RR], mean difference [MD] or standardised mean difference [SMD]). In case of discrepancy or missing information in the published meta-analyses, original clinical trials were retrieved for clarification.

We re-analysed all meta-analyses and expressed the results using Hedges' g standardised mean difference (SMD) and 95% confidence intervals (95% CI). We used random-effects models and Restricted Likelihood Maximum (“REML”) estimators. When different estimates were used for the same intervention-outcome combination (e.g. odds ratio [OR] and mean difference [MD]), separate pairwise meta-analyses were performed, then results were back transformed to SMD and meta-analysed to get pooled estimates.^31^ In the presence of multiple effect sizes per study (e.g. several doses or QoL tools), a unique effect size per study was calculated using the Borenstein's approaches.^32^ We also re-estimated the results of meta-analyses using MD for continuous outcomes and OR for dichotomous outcomes to improve clinical interpretation of the findings. To ensure that the estimates were consistent across studies, we carefully reviewed each RCT individually to determine exactly what statistical measures were reported. When necessary, we applied validated transformation methods^33^^,^^34^ to convert median and interquartile range or standard error values into mean (standard deviation [SD]) values, as detailed in Table 1 Legend. The results for QoL were back-transformed from pooled SMD to Short Form-36 scores using the mean baseline SD of included studies.^31^

**Table 1:** Baseline data from the randomized controlled trials from the included meta-analyses.

Meta-analysis author, year	Protocol registration	Databases searched (dates)	RCTs included in the umbrella, n	Sample size, n	Sex^a^, n (%)	Mean				Intervention	Comparator(s)	Main outcomes
						Age, y	AHI, /h	BMI, kg/m^2^	ESS score			
Alrubasy et al. 2025^35^	CRD42024538949	PubMed, Web of Science, ScienceDirect, Cochrane Central Register of Controlled Trials (CENTRAL), and ClinicalTrials.gov March 1, 2024	3	273	232 (82)	56.1 (9.5)	23.7 (15)	28.1 (3.6)	10.1 (4.7)	HNS	Inactive	AHI ESS AE
Altobaishat et al. 2025^36^	CRD42024562853	PubMed, EMBASE, Cochrane Library, Scopus, and Web of Science Up to 24 June 2024	3	828	585 (71)	49.2 (10.7)	50 (27.1)	39.0 (6.5)	10.2 (5.1)	GLP-1: Liraglutide Tirzepatide	Placebo	AE
Brill et al. 2017^37^	NR	MEDLINE, Embase, Cochrane Library From 1980–Nov 2016	8	472	282 (60)	66.8 (12)	29.8 (18.8)	27.9 (4.5)	6.9 (3.7)	CPAP	Standard care Sham CPAP	AHI Adherence
Carneiro-Barrera et al. 2019^38^	CRD42018102740	CINAHL, ProQuest, Psicodoc, Scopus, Web of Science From inception to Apr 2018	7	643	360 (60)	56.4 (9.6)	32.7 (13.8)	33.3 (4.5)	10.8 (4.8)	Physical activity Diet CPAP MAD	Lifestyle modification Usual care Physical activity Sleep hygiene	AHI ESS score
De Vries et al. 2017^39^	NR	PubMed, Embase, CINAHL Up to 31 Dec 2016	5	319	246 (77)	48.7 (11)	23.2 (12)	29.1 (4.2)	11.8 (2.3)	MAD	CPAP Conservative measures Placebo No treatment	BP
Ferreira et al. 2025^17^	CRD42020159132	PubMed, EMBASE, The Cochrane Central Register of Controlled Trials (CENTRAL), the (LILACS), Healthy Cities (CidSaúde), the PAHO, the REPIDISCA, the Nursing Database (BDENF), the Caribbean Health Sciences Literature, the WHOLIS, the IBECS and the SciELO Until December 2020	3	162	53.7 (12)	34.2 (15.9)	29.1 (5.4)	29.9 (5.1)	10.9 (5.1)	OMT CPAP Physical activity	Inactive CPAP	AHI ESS
Edwards et al. 2012^40^	NR	CINAHL, Cochrane library, Embase, OVID Medline, Scopus (up to May 2018)	1	32	32 (100)	49.1 (8.3)	41.6 (22.1)	28.3 (2.6)	13.4 (4.5)	Physical activity Diet Physical activity + diet CPAP	No treatment Sleep hygiene Stretching CPAP Diet	AHI
Gao et al. 2019^41^	NR	PubMed, EMBASE, Cochrane library, Cochrane Database of Systematic Reviews From inception to 9 Aug 2016	46	2341	1851 (79)	49.3 (10.3)	33.9 (18.6)	30.3 (5.5)	11 (4.8)	CPAP Physical activity MAD Oral surgery Positional therapy Oxygen therapy Lifestyle modification Oral surgery + MAD	Sham No treatment MAD CPAP Oral surgery Positional therapy Physical activity	AHI ESS score
Gao et al. 2025^42^	NR	PubMed, Scopus, Web of Science, Cochrane Central Register of Controlled Trials (CENTRAL), clinicaltrials.gov, and Google Scholar From inception to 4 June 2024	14	1141	791 (73)	50 (10.9)	20.9 (10.8)	28.3 (4.9)	9.9 (6.9)	PT MAD	Placebo Inactive No treatment PT MAD	AHI ESS QoL AE
He et al. 2018^43^	NR	PubMed+, Embase, Web of Science, Cochrane Library Up to 1 Aug 2017, updated to Dec 25, 2018	1	72	72 (100)	46.7 (NR)	19 (8.8)	28.3 (4.9)	NR	Oral surgery	MAD	AHI ESS score
Kang et al. 2002^44^	CRD42020154425	PubMed, MEDLINE, EMBASE, Cochrane From inception to Mar 2020	2	111	100 (90)	47.1 (12.3)	43.5 (18.4)	27.9 (2.8)	12 (4.4)	Oral surgery HNS	Inactive	BP
Kou et al. 2022^45^	CRD42021240891	PubMed, EMBASE, Web of Science, Cochrane Library From inception to 1 Jun 2021	1	27	13 (48)	45.8 (8.7)	28.3 (38.5)	38.1 (5.9)	NR	CPAP MAD Nocturnal supplemental oxygen Oral surgery Drug treatments Bariatric surgery	Inactive Drug treatments	BP
Kovacs et al. 2022^46^	CRD42019138998	Medline, Embase, CENTRAL, Scopus From inception to 29 May 2020	8	907	564 (62)	47 (10.2)	33.6 (27.6)	36.5 (11.7)	11.2 (4.6)	CPAP Diet CPAP + diet	Diet CPAP	BP
Li M et al. 2025^25^	NR	PubMed and Web of Science Until July 1, 2024	5	947	672 (71)	49.8 (3.1)	48.1 (11.2)	37.6 (4.1)	10.1 (2.5)	GLP-1: Tirzepatide Liraglutide	Placebo	BP
Li Z et al. 2022^47^	NR	PubMed, EMBASE, Cochrane Library From Jan 1994 to Oct 2021	41	7332	5550 (76)	57.1 (11.5)	31.2 (22.6)	31.1 (6.9)	11.5 (12.2)	CPAP	Usual care Sham CPAP Placebo No treatment Conservative measures	ESS score
Lins-Filho et al. 2020^48^	CRD42019127970	PubMed, Medline, Scopus, Cochrane Controlled Registry of Trials	4	180	88 (56)	51.67 (9)	29.27 (22)	30.55 (6.4)	9.8 (4.7)	Physical activity	No treatment Stretching Health education	AHI QoL (SF-36) ESS score
Lins-Filho et al. 2021^49^	CRD42020210509	PubMed, Medline, Scopus, Cochrane Controlled Registry of Trials	2	162	100 (62)	62.5 (5.4)	21.7 (5.9)	28.5 (3.4)	7.9 (4.4)	Physical activity	No treatment Health education Diet Diet + physical activity	AHI
Locke et al. 2025^50^	CRD42022378853.	PubMed, Cochrane CENTRAL, EMBASE From inception to October 1, 2022.	1	49	28 (53)	48.6 (9.8)	49.8 (27.2)	38.9 (3)	10.1 (4.6)	WL (BS)	CPAP	AHI
Lv et al. 2024^51^	CRD42023456380	PubMed, Web of Science, EMBASE, Cochrane Library, and Scopus Up to December 3, 2023.	29	5231	3652 (70)	61.3 (9.2)	33.7 (17.2)	31.3 (5.2)	8.7 (4.2)	CPAP	Inactive	BP
Revuelta et al. 2024^52^	CRD42020192179	PubMed, Scopus, Cochrane, and Web of Sciences Up to March 2024.	12	527	333 (63)	51.7 (10.7)	29.5 (14.5)	30.8 (4.7)	9.7 (4.9)	Physical activity WL (Diet)	No treatment WL (Diet)	
Mohamed et al. 2024^53^	CRD42024517491	SCOPUS, PubMed, Cochrane Library, Web of Science Until Dec 2023	3	258	191 (74)	46.5 (11.3)	14.0 (7.2)	26.9 (3.7)	8.7 (5)	MAD	PT	AHI ESS score QoL (FOSQ)
Patil et al. 2019^7^	NR	NR	38	6839	4750 (70)	51.5 (10.3)	21.1 (31)	33.8 (6.7)	15.1 (5.7)	CPAP	Inactive	AHI ESS score QoL BP Adherence
Pepin et al. 2024^23^	CRD42023434640	PubMed, Embase, clinicaltrials.gov Up to 12 Jun 2024	20	4029	2980 (74)	51.4 (10)	17.6 (30)	33.7 (6.8)	15.0 (3.8)	Pitolisant Solriamfetol Modafinil	Placebo	ESS score QoL Safety
Rangarajan et al. 2022^54^	CRD42021193386	Google Scholar, Cochrane Trial Registry, PubMed, LILACS, Ovid	3	273	207 (76)	49.0 (10.5)	23.6 (16.5)	28.7 (4.2)	11.4 (3.6)	MAD	CPAP Placebo No treatment MAD Physical activity Positional therapy	QoL (FOSQ, SF-36, SAQLI)
Rueda et al. 2020^18^		Cochrane Airways Trials Register, the Cochrane Register of Studies Cochrane Central Register of Controlled Trials (CENTRAL), via All years to 1 May 2020) MEDLINE Ovid SP (1946–1 May 2020) Embase Ovid SP (1974–1 May 2020)	1	100	100 (100)	48.1 (11.2)	30.9 (20.6)	27.4 (4.9)	12.7 (3.0)	OMT	CPAP	ESS AHI
Schwartz et al. 2018^55^	NR	Medline through PubMed, Web of Science, Cochrane Library (up to 12 Jul 2016; updated 30 Mar 2017)	7	566	463 (82)	47.7 (9.9)	28.1 (18.7)	29.7 (6.5)	11.3 (4.5)	MAD	CPAP Placebo	AHI ESS QoL (FOSQ, SF-36) Adherence
Silva De Sousa et al. 2023^56^	CRD42018096980	Pubmed, Embase Cochrane Central Register of Con-trolled Trials (CENTRAL), Latin American and Caribbean Literature on Health Sciences (LILACS) July 2022	4	120	82 (68)	60.4 (8.8)	28.8 (11.7)	28.9 (4.7)	11.1 (5.8)	OMT	Inactive	BP
Tang et al. 2024^57^	CRD 42024501348	PubMed, Embase and Web of Science Until January 20	15	729	523 (72)	60.6 (10.7)	27.4 (13.2)	28.9 (4.3)	9.8 (4.9)	Physical activity OMT	Inactive	AHI ESS
Vimal et al. 2022^58^	CRD42020131068	Medline, Embase From 1946 onwards	3	207	174 (84)	52.4 (10.4)	36.4 (20)	27.2 (3.3)	9.9 (4.4)	MAD	CPAP Sham	AHI ESS Adherence BP
Wollny et al. 2024^59^	NR	Medline, Embase, Cochrane, and Google Scholar January 2000–December 2023	1	138	119 (86)	55.5 (9.1)	37.9 (9.8)	29.8 (3.0)	11.4 (4.8)	HNS	Inactive	AE
Wong et al. 2018^60^	CRD42017062359	Embase, Ovid, PubMed, Cochrane Library Review and ClinicalKey Up to 18 Dec 2017	2	120	74 (62)	45.5 (10.5)	39.9 (33.4)	45.8 (6.2)	8.5 (5.7)	Bariatric surgery	CPAP Diet	AHI
Yang R et al. 2025^61^	CRD42024558287	Web of Science, Scopus, PubMed, APA PsycInfo, Embase, Ovid, Cochrane Library, CINAHL, Clinicaltrials.gov, and International Clinical Trials Registry Platform (ICTRP) Up to May 22, 2024	3	478	345 (72)	49.8 (9.4)	45.7 (25.5)	36.6 (7.8)	9.8 (5.3)	GLP-1: Liraglutide Tirzepatide GLP-1+CPAP	Placebo CPAPA	AHI
Zhang et al. 2019^62^	NR	PubMed, ISI Web of Knowledge, Ovid, EBSCO Dentistry & Oral Science Source, Cochrane Library, Embase Up to 23 May 2017	13	740	618 (84)	48.1 (10.2)	27.8 (17.3)	31.2 (6.4)	10.7 (4.4)	MAD	CPAP	AHI QoL (FOSQ, SF-36) ESS score
Zhou et al. 2021^63^	CRD42020152077	Medline, Embase (up to 6 May 2020)	1	50	43 (86)	49.1 (9.1)	56.8 (16.5)	32.7 (5.8)	11.6 (2.8)	Oral surgery	CPAP Inactive	AHI ESS score

Values are descriptive statistics derived from the randomised controlled trials (RCTs) included in our meta-analysis. To ensure that the estimates were consistent across studies, we carefully reviewed each RCT individually to determine exactly what statistical measures were reported. When necessary, we applied validated transformation methods^34^^,^^64^ to convert median and interquartile range or standard error values into mean (standard deviation values). The values presented in the table are therefore weighted means (weighted standard deviations), calculated using the sample size of each study as weights using the formula.

AHI, apnoea–hypopnea index; BMI, body mass index; BP, blood pressure; CPAP, continuous positive pressure; ESS, Epworth Sleepiness Scale; FOSQ, Functional Outcomes of Sleep Questionnaire; HNS, hypoglossal nerve stimulation; NR, not reported; OSF, Open Science Framework; QoL, quality of life; RCT, randomised controlled trial; SAQLI, Sleep Apnoea Quality of Life; SF36, Short Form-36 questionnaire.

$W\text{eighted}\;\text{Mean}=\frac{\sum_{i=1}^{n}\left(xi*wi\right)}{\sum_{i=1}^{n}wi}$

${SD}_{W}=\sqrt{\frac{\sum_{i=1}^{n}w_{i}{\left(x_{i}\;-\;{\bar{x}}_{w}\right)}^{2}}{\frac{\left(M\;-\;1\right)}{M}\sum_{i=1}^{n}w_{i}}}$

a Sex (male %) refers to biological sex, as reported in the original studies.

We also recalculated the between-study heterogeneity using the I^2^ statistic and 95% confidence intervals. Additionally, we assessed small study effect using the Egger regression asymmetry test (where a P value ≤ 0.10 indicates publication bias) and excess significance bias^64^; this test was only used for meta-analyses that included ≥10 RCTs. Statistical analyses were performed with R (4.1.1) and metaumbrella and meta packages.

We assessed the methodological quality of the included meta-analyses using the AMSTAR 2 tool. This consists of a 16-item checklist where each item was initially rated as *Yes*, *Partial Yes*, or *No*, as per the suggested checklist responses. To simplify interpretation and facilitate comparison across studies, we applied a dichotomized scoring system in which only items rated as *Yes* (indicating full adherence to the criterion) were classified as meeting each criteria. Both *Partial Yes* (reflecting partial adherence) and *No* (non-adherence) ratings were grouped together as ‘not fully fulfilled.’ This dichotomization allowed us to generate a clear overview of methodological rigor across the included meta-analyses. Seven items (2, 4, 7, 9, 11, 13, and 15) are considered as critical.^65^^,^^66^ The meta-analyses were categorised as high, moderate, low, or critically low quality. The criteria for these ratings are detailed in Supplementary Appendix Table D. Two authors (CF and RBM) independently rated the overall quality; in cases of disagreement, consensus was reached by consulting additional authors (CK and JLP).

The certainty of evidence from meta-analyses of RCTs was classified as high, moderate, low, or very low, based on GRADE.^67^ The level of certainty was determined based on five domains: the risk of bias, inconsistency, indirectness, imprecision and publication bias. We extracted assessment of risk of bias from meta-analyses if authors used the Cochrane risk-of-bias tool. If other tools were used, we reassessed the risk of bias of each original study in the meta-analyses using the Cochrane risk-of-bias 2 tool.^68^

We downgraded the quality of evidence for risk of bias when <75% of the included RCTs were assessed as having a low risk of bias (serious concern). We applied a very serious downgrade when <50% of the RCTs met this criterion. We downgraded for inconsistency when I^2^ values were >75% and, for very serious inconsistency if I^2^ values were >90%. For indirectness, we downgraded if studies mixed sleep-disordered breathing populations or different types of control groups. We downgraded the certainty of evidence for serious imprecision if the 95% CI value of the SMD had a width >0.8, and for very serious imprecision if the lower and upper boundaries of SMD 95% CI value crossed both important effect size boundaries (−0.8 and 0.8). We downgraded for publication bias if Egger regression or excess of significant bias tests were significant. We upgraded evidence by one level if the SMD was above or below 0.8 (large effect size) and by two levels if the SMD was above or below 1.2. Starting from high, the level of evidence was downgraded to moderate if there were one or two downgrades, to low with three or four downgrades, and to very low with five or six downgrades.^41^ The GRADE assessment was conducted independently by two authors (CF and RBM) and final decision was reached by a third author (CK).

### Role of the funding source

This study did not receive any external funding. Supporters of individual authors had no role in study design, data collection, data analysis, data interpretation, or writing of the report. All authors had full access to the study data. JLP and CK assumed final responsibility for the decision to submit the manuscript for publication.

## Results

### Literature identification and selection

**Fig. 1:**
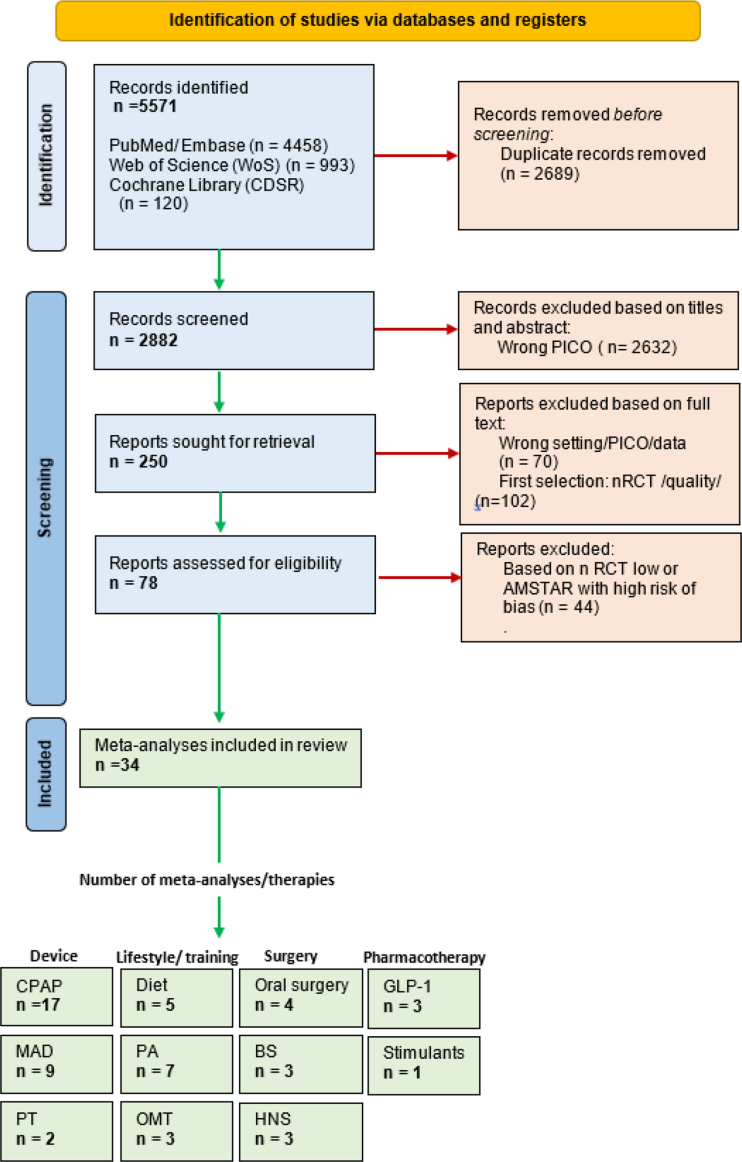
Flowchart of the umbrella meta-analysis. AMSTAR 2, Assessment of Multiple Systematic Reviews 2; BS, Bariatric surgery; CPAP, continuous positive airway pressure; GLP-1, Glucagon-like peptide-1; HNS, Hypoglossal nerve stimulation; MAD, mandibular advancement device; OMT, Orofacial myofunctional therapy; PA, Physical activity; PT, Positional therapy; RCT, randomised controlled trials.

A total of 5571 records were retrieved through literature search and 5321 articles, including 2689 duplicates, were excluded. After reviewing the remaining 250 full-text articles, we excluded 172 articles and selected 78 for eligibility assessment. Thirty-four meta-analyses, analysing 230 RCTs published between 1996 and 2024 featuring multiple interventions and control groups were included in the quantitative synthesis (Fig. 1).

The interventions (number of meta-analyses) evaluated in this umbrella review included CPAP (n = 17; 50%),^7^^,^^17^^,^^25^^,^^36^, ^37^, ^38^^,^^40^, ^41^, ^42^^,^^46^^,^^47^^,^^50^^,^^51^^,^^55^^,^^61^, ^62^, ^63^ MAD (n = 9; 27%),^39^^,^^41^, ^42^, ^43^^,^^53^, ^54^, ^55^^,^^58^^,^^62^ physical activity (n = 7; 21%),^17^^,^^40^^,^^41^^,^^48^^,^^49^^,^^52^^,^^57^ behavioural weight loss management (diet [n = 5; 15%])^38^^,^^46^^,^^48^^,^^52^^,^^60^ or bariatric surgery [n = 3; 6%]^45^^,^^50^^,^^60^ positional therapy (n = 2; 6%),^42^^,^^53^ GLP-1 agonists (n = 3; 9%),^36^^,^^51^^,^^61^ wake stimulants (n = 1; 3%),^23^ HNS (n = 3; 9%),^35^^,^^44^^,^^59^ myofunctional therapy (n = 3; 9%),^17^, ^18^, ^56^, ^57^ and oral surgery (n = 4; 12%).^41^^,^^43^^,^^44^^,^^63^ The main characteristics of the included meta-analyses are summarised in Table 1.

A total of 36,353 participants were included, of whom 26,058 (72%) were male. The mean (SD) age was 54.0 (11.3) years, the mean (SD) body mass index (BMI) was 32.2 (6.8) kg/m^2^, the mean (SD) apnoea–hypopnoea index (AHI) was 28.7 (24.7) events per hour, and the mean (SD) Epworth Sleepiness Scale (ESS) score was 11.8 (7.4).

The AMSTAR 2 score of included meta-analyses ranged from 6 to 15. Overall, the quality was high for three meta-analyses,^23^^,^^24^^,^^50^ moderate for five meta-analyses,^42^^,^^53^^,^^54^^,^^60^^,^^62^ low for three meta-analyses,^18^^,^^38^^,^^49^ and critically low for the majority of meta-analyses^7^^,^^17^^,^^25^^,^^35^^,^^36^^,^^39^^,^^41^, ^42^, ^43^, ^44^^,^^46^, ^47^, ^48^^,^^50^, ^51^, ^52^^,^^56^, ^57^, ^58^, ^59^, ^60^, ^61^ (see Table E in the Supplementary Appendix for full details). The most common limitations were that authors did not provide the list of excluded studies, search strategy justification, or details of funding.

### Summary of evidence for the different health outcomes

#### Apnoea–hypopnoea index (AHI)

**Fig. 2:**
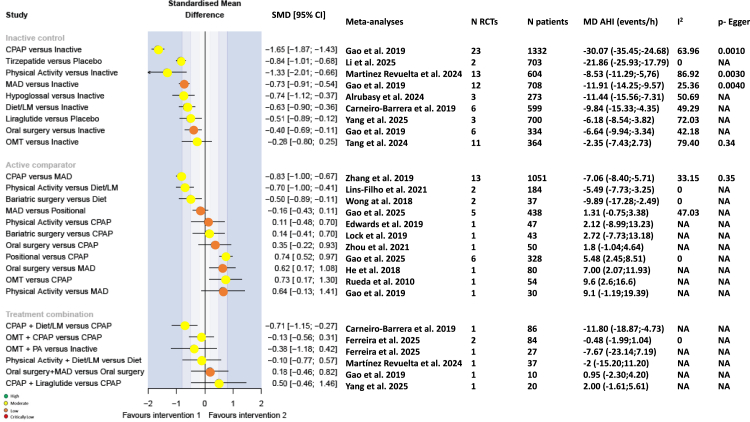
Forest plots for the apnoea–hypopnoea index endpoint. CI, confidence interval; CPAP, continuous positive airway pressure; e, exponential; I^2^, evaluation of heterogeneity; LM, lifestyle measures; MAD, mandibular advancement device; MD, mean difference; NA, not applicable; SMD, standardised mean difference.

SMD values for the effects of interventions and combinations of interventions versus comparators on the AHI are shown in Fig. 2. Compared with any inactive control, CPAP was the most effective treatment for reducing the AHI (MD –30.7 events/h; SMD –1.65, 95% CI –1.87, −1.43). Among emerging pharmacologic therapies, GLP-1 receptor agonists showed promising results. The SMD for tirzepatide, a dual gastric inhibitory polypeptide (GIP)/GLP-1 receptor agonist, was −0.84 (95% CI –1.68, −1.14), with a mean AHI reduction of −21.85 events/h. Similarly, liraglutide, a long-acting GLP-1 receptor agonist, was associated with a significant reduction in AHI (SMD –0.51, 95% CI –0.89, –0.12; MD –6.18 events/h). Non-pharmacological interventions such MAD, physical activity and diet also improved the AHI, though to a lesser extent than CPAP with SMD (95% CI) values compared with inactive control of −0.73 (−0.91, −0.54), −1.33 (−2.01, −0.66) and −0.63 (−0.90, −0.36), respectively, and MD values of −11.91, −8.53, and −9.84 events/h, respectively. HNS did not have a statistically significant effect on the AHI, with marked heterogeneity between studies (SMD –0.74, 95% CI –1.12, −0.37) and a MD of −11.44 events/h.

CPAP was superior to a MAD for reducing the AHI (MD –7.06 events/h; SMD –0.83, 95% CI –1.00, −0.67). CPAP was also superior to positional therapy (MD –5.48 events/h) but this result was not statistically significant. Physical activity was more effective than diet (MD –5.49 events/h; SMD –0.7, 95% CI –1.00, −0.41), and bariatric surgery was more effective than diet alone (MD –9.89 events/h; SMD –0.5, 95% CI –0.89, −0.11). Conversely, oral surgery was less effective at improving the AHI than a MAD (MD +7.00 events/h; SMD 0.62, 95% CI 0.17, 1.08).

Combining weight loss management strategies (diet plus lifestyle modifications) with CPAP was more effective at reducing the AHI than CPAP alone (SMD –0.71, 95% CI –1.15, −0.27). However, adding a MAD to oral surgery did not reduce the AHI to a significantly greater extent than oral surgery alone.

#### Excessive daytime sleepiness (ESS score)

**Fig. 3:**
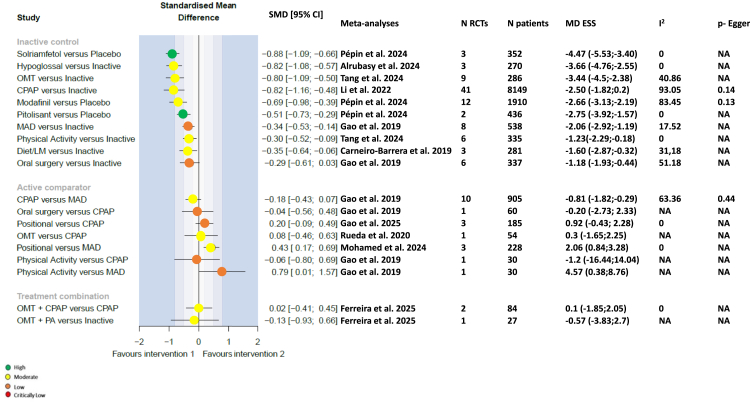
Forest plots for the Epworth Sleepiness Scale score endpoint. CI, confidence interval; CPAP, continuous positive airway pressure; e, exponential; I^2^, evaluation of heterogeneity; MAD, mandibular advancement device; MD, mean difference; NA, not applicable; SMD, standardised mean difference.

SMD values for the effects of interventions and combinations of interventions versus comparators on the ESS score are shown in Fig. 3. Compared with inactive control, all interventions apart from physical activity (SMD –1.76, 95% CI –3.71, 0.20) and oral surgery (SMD –0.29, 95% CI –0.61, 0.03) effectively reduced the ESS score (there was marked heterogeneity between studies reporting the effects of physical activity). CPAP, the wakefulness-promoting agents solriamfetol, pitolisant and modafinil, and HNS were the most effective treatments at reducing the ESS score (MD –2.5, −4.47, −2.75, −2.66 and −4.4, respectively, with corresponding SMD [95% CI] values versus placebo of −0.82 [–1.16, −0.48], −0.88 [–1.09, −0.66], −0.51 [–0.73, −0.29], −0.69 [–0.98, −0.39], and −0.88 [–1.5, −0.26]).

A MAD was more effective than positional therapy at reducing the ESS (SMD –0.43, 95% CI –0.69, −0.17). CPAP was not superior to physical activity (SMD –0.18, 95% CI –0.43, 0.07), oral surgery (SMD –0.04, 95% CI –0.56, 0.48), or positional therapy (SMD 0.19, 95% CI –0.21, 0.60) for improving daytime sleepiness in direct comparisons, but these data came from a limited number of small trials, and therefore certainty of evidence is very low. There were no direct comparisons between wakefulness-promoting agents and other active interventions.

#### Health-related quality of life (QoL)

**Fig. 4:**
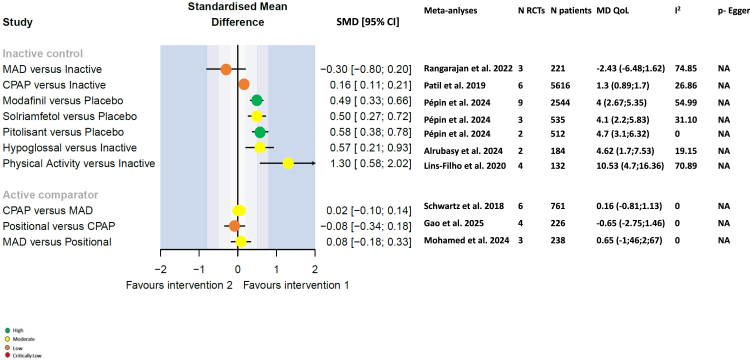
Forest plots for quality-of-life measure endpoints (including the Functional Outcomes of Sleep Questionnaire, Sleep Apnoea Quality of Life Index, Short Form-36, Clinical Global Impression scale, and Patient Global Impression scale). CI, confidence interval; CPAP, continuous positive airway pressure; e, exponential; I^2^, evaluation of heterogeneity; MAD, mandibular advancement device; MD, mean difference; NA, not applicable; SMD, standardised mean difference.

SMD values for the effects of interventions and combinations of interventions versus comparators on health-related QoL are shown in Fig. 4. CPAP was associated with a modest but statistically significant improvement compared with inactive intervention for this endpoint (SMD 0.16, 95% CI 0.11, 0.21). Stimulants (modafinil, solriamfetol and pitolisant) improved QoL to a similar extent (SMD [95% CI] values of 0.49 [0.33, 0.66], 0.50 [0.27, 0.72] and 0.58 [0.38, 0.78], respectively). The largest effect size was seen for physical activity compared with an inactive comparator, with a SMD of 1.30 (95% CI 0.58, 2.02). In contrast, head-to-head comparisons between active treatments revealed minimal differences: CPAP and MAD showed nearly equivalent effects on QoL (SMD 0.02; 95% CI –0.10, 0.14), as did MAD and positional therapy (SMD 0.008; 95% CI –0.18, 0.33). These findings highlight the comparatively greater impact of stimulants and physical activity on QoL, while suggesting that differences between standard active therapies may be marginal.

#### Blood pressure (BP)

**Fig. 5:**
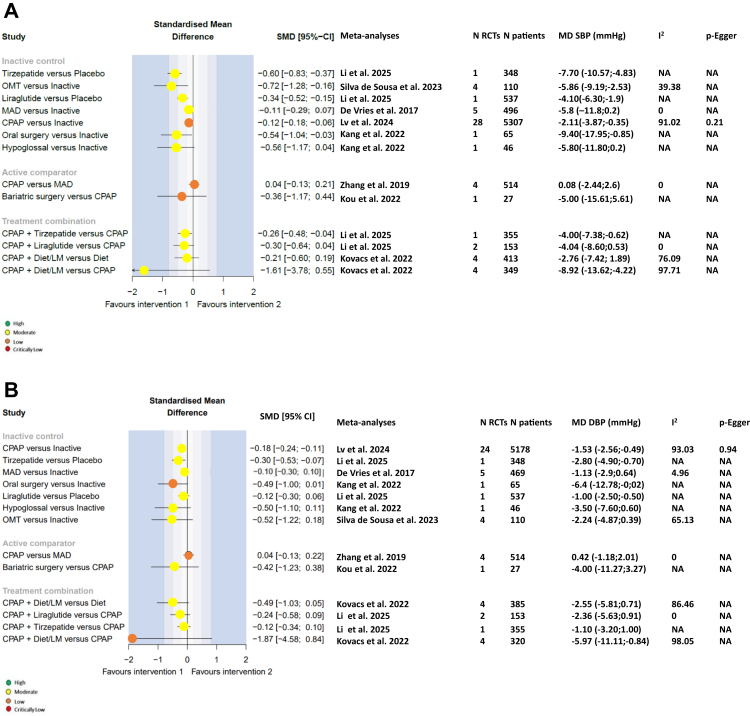
Forest plots for blood pressure endpoints (**A**. Office systolic blood pressure; **B**. office diastolic blood pressure). CI, confidence interval; CPAP, continuous positive airway pressure; e, exponential; I^2^, evaluation of heterogeneity; LM, lifestyle measures; MAD, mandibular advancement device; MD, mean difference; NA, not applicable; SMD, standardised mean difference.

SMD values for the effects of interventions and combinations of interventions versus comparators on BP are shown in Fig. 5. Compared with an inactive intervention, treatment with a GLP-1 agonist such as tirzepatide markedly reduced systolic BP (MD –7.7 mmHg; SMD –0.6, 95% CI –83, −0.37, underscoring the emerging role of these agents in cardiovascular risk management beyond glucose control. The most effective traditional interventions that for reducing office systolic BP (Fig. 5A) were HNS (MD –5.8 mmHg; SMD –0.56, 95% CI –1.17, −0.04) and oral surgery (MD –9.40 mmHg; SMD –0.54, 95% CI –1.04, −0.03). However, these findings derive from a single study (n = 65) each, limiting generalisability. CPAP and oral surgery also lowered office diastolic BP (Fig. 5B) (MD –1.94 and −6.4 mmHg, respectively; corresponding SMDs of −0.18 [95% CI –0.24, −0.11] and −0.49 [95% CI –1.00, 0.01]). No other interventions significantly reduced office BP compared with an inactive control, and CPAP was not superior to MAD or bariatric surgery for reducing systolic and diastolic BP. In addition, combining weight loss interventions with CPAP did not reduce office BP to a significantly greater extent than CPAP or diet alone. Effects on BP evaluated using ambulatory BP monitoring have only been investigated for CPAP.^2^ This meant that 24-h BP was not included because this endpoint has only been studied in RCTs of CPAP treatment in our meta-analysis.

#### Major adverse cardiovascular events (MACE)

MACEs were not included because this endpoint has only been studied in RCTs of CPAP treatment.

#### Adherence

**Fig. 6:**
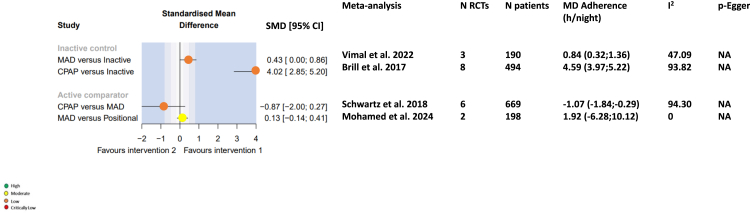
Forest plots for adherence endpoints. CI, confidence interval; CPAP, continuous positive airway pressure; e, exponential; I^2^, evaluation of heterogeneity; MAD, mandibular advancement device; MD, mean difference; NA, not applicable; SMD, standardised mean difference.

SMD values for adherence during the use of MAD and CPAP versus inactive comparators, and for comparisons between interventions are shown in Fig. 6. Compared with an inactive comparator (sham), short-term adherence was better during treatment with MAD or a CPAP (SMD [95% CI] 0.43 [0, 0.86] and 4.02 [–2.85, 5.20], respectively). Adherence (device usage) was not different with a MAD compared with CPAP (SMD –0.87, 95% CI –2.00, 0.27), and there was no significant difference in adherence to MAD compared with positional therapy (SMD 0.20, 95% CI –0.08, 0.48).

#### Safety

**Fig. 7:**
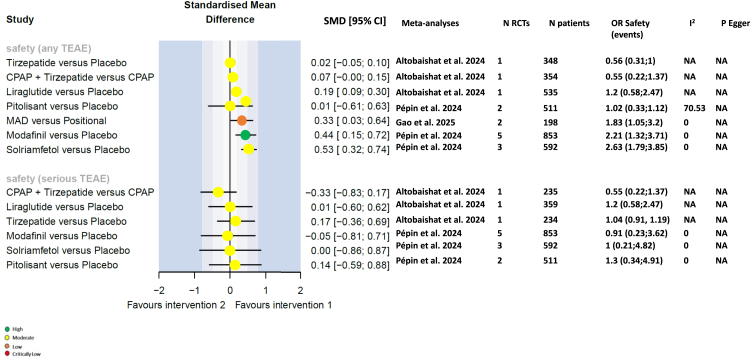
Forest plots for safety endpoints. CI, confidence interval; e, exponential; I^2^, evaluation of heterogeneity; NA, not applicable; OR, odds ratio; SMD, standardised mean difference; TEAE, treatment-emergent adverse event. For the forest plots, green circle for high, yellow circle for moderate, orange circle for low and red circle for critically low certainty of evidence.

Only the meta-analysis of stimulants reported adverse events compared with an inactive control group (placebo); some other meta-analyses did report safety data but a comparison between the treatment and control groups. The rate of adverse events was similar with pitolisant compared with placebo, but adverse events were more common during treatment with modafinil and solriamfetol than with placebo (SMD [95% CI] 0.44 [0.15, 0.72] and 0.53 [0.32, 0.74], respectively) (Fig. 7).

### Certainty of evidence (GRADE assessment)

The certainty of evidence for each treatment-outcome result was assessed using the GRADE classification. The ratings showed that 12 studies (35%) had evidence that was of “low” certainty,^7^^,^^37^^,^^40^, ^41^, ^42^^,^^46^,^49^, ^50^, ^51^, ^52^^,^^55^^,^^62^^,^^63^ 23 studies (68%) had “moderate” certainty of evidence,^18^^,^^23^^,^^25^^,^^35^^,^^36^^,^^38^^,^^39^^,^^41^^,^^42^^,^^44^^,^^47^, ^48^, ^49^, ^50^^,^^52^^,^^54^, ^55^, ^56^, ^57^, ^58^^,^^60^, ^61^, ^62^ and only one study (3%) provided evidence that was of “high” certainty^3^ (see Table F in the Supplementary Appendix for full details).

## Discussion

This umbrella review presents a comprehensive landscape of OSA therapy, summarising the efficacy of conventional and emerging treatments such as CPAP, MAD, diverse weight loss strategies, muscle stimulation (oropharyngeal therapy, HNS) and pharmacological agents. Including a wide range of therapies is uncommon in the literature. The key findings of this umbrella review of meta-analyses of RCT data evaluating the effects of OSA treatments on a variety of clinically relevant outcomes are as follows. CPAP was superior to any other intervention for reducing the AHI and was particularly effective when combined with weight loss management. However, a MAD, physical activity and weight loss also had a moderate clinically relevant effect on the AHI. Bariatric surgery was substantially more effective for reducing the AHI than lifestyle interventions. CPAP and wake stimulants were the most effective interventions for improving daytime sleepiness, while a MAD and diet had moderate effects. Physical activity was by far the most effective intervention with respect to improvements in QoL, followed by CPAP and stimulants. Both CPAP and a MAD reduced BP to a similar extent. In addition to these conventional therapies, emerging strategies such as GLP-1 agonists are gaining attention as promising future therapies. Originally developed for the management of obesity and type 2 diabetes, GLP-1 agonists have recently been tested in populations with both obesity and OSA, showing encouraging results in reducing OSA severity through weight loss and possibly other mechanisms. Finally, we identified significant knowledge gaps regarding non-CPAP therapies for OSA (except MAD), a lack of direct comparisons between treatment options, a lack of data regarding combination therapy for OSA, and a lack of comparative safety data for the different treatments.

Overall, CPAP and MADs were the most effective treatments for improving OSA severity and symptoms. However, the ongoing effectiveness of CPAP is limited by difficulties in achieving adequate long-term adherence and therapy continuation.^8^^,^^9^^,^^69^ Although comparative safety data are scarce, the main limitation of MAD therapy is likely to be its tolerability and long-term dental and temporomandibular joint side effects^70^^,^^71^ Our umbrella review data did not achieve statistical significance with respect to better mid-term adherence to a MAD but this is probably due to heterogeneity of the studies in the selected meta-analysis.^55^ In contrast, a recently published RCT with objective measure of MAD adherence showed that despite the higher efficacy of CPAP on AHI and higher adherence to MAD, both demonstrate comparable clinical effectiveness on patient-centred outcomes.^72^ Overall, the two treatments appear to be equivalent in terms of their impact on patient-centred outcomes and reductions in BP, meaning that the choice of an MAD or CPAP for a given patient is likely to depend on the presence of any dental or joint contraindications for MAD therapy, the degree of obesity, and patient preference.^13^^,^^73^, ^74^, ^75^

Nearly half of all people with OSA started on CPAP have stopped therapy after 3 years.^8^ However, there is not currently any clearly defined strategy for the management of CPAP therapy termination. The clinically relevant, if moderate, effect of a MAD, physical activity and weight loss on the AHI mean that these interventions could be of value for individuals with mild to moderate OSA or those who have refused or terminated CPAP therapy. It has recently been shown that restarting and continuing CPAP after initial therapy termination is beneficial, with a 40% reduction all-cause mortality seen in those restarting and continuing CPAP in observational studies.^5^ In addition, the choice of management strategy after CPAP therapy termination might be influenced by the main goal of therapy. If reducing daytime sleepiness is the goal then restarting CPAP, using a MAD or wake stimulants are the best potential strategies. To obtain the greatest improvements in quality of life, recommending physical activity would be the preferred option.

For patients with less severe OSA, especially those with impaired QoL and mild or moderate AHI, the use of a MAD and lifestyle measures might be a reasonable strategy. This group of individuals generally has little or no cardiovascular risk and therefore treatments targeting patient-reported outcome measures are most appropriate. In that context, prioritising and promoting physical activity would be a good strategy to improve QoL in patients with mild to moderate OSA. The value of physical activity has traditionally been underestimated in patients with OSA at low to medium cardiovascular risk. However, physical activity was found to result in by far the greatest QoL improvements in the current analysis, along with reducing daytime sleepiness and moderate improvements in the AHI. These findings suggest that physical activity should be more widely incorporated into OSA management strategies, either alone or in combination with more specific therapies.

In OSA with daytime sleepiness, CPAP remains the first-choice treatment due to its ability to improve the ESS score. A MAD had a statistically significant but more moderate effect on daytime sleepiness, while wake stimulants could help to ameliorate symptoms (although they do not directly target upper airway obstruction and therefore do not treat the underlying cause of OSA). Although pitolisant had a similar tolerability profile to placebo in the current analysis, it is important to note that the safety of wake-promoting agents may be different in patients with OSA who have comorbidities and are at high cardiovascular risk.^76^ Individuals with OSA and a high comorbidity burden who are minimally asymptomatic are probably good candidates for intensive physical activity and weight loss programmes. However, the relationship between CPAP adherence and adherence to healthy behaviours, including medication use, remains unclear. Recent evidence suggests that patients who maintain healthier behaviours are more likely to adhere to CPAP therapy over time.^77^ Nonetheless, given that some patients demonstrate poor CPAP adherence, achieving sustained compliance with physical activity and lifestyle interventions remains challenging, underscoring the need for integrated support strategies. RCT data indicate that patients with OSA who stopped an exercise programme after 9 months had worse OSA severity during longer-term follow-up than those who continued to exercise after the 9-month duration of the RCT intervention.^78^ The AHI and oxygen desaturation index benefits obtained during the exercise programme declined over another year of follow-up, but the beneficial effects of exercise on daytime sleepiness and mental health persisted.^78^

This umbrella review provides some clarity around the place of weight loss strategies in the management of OSA. Bariatric surgery was more effective than diet at reducing the AHI. However, bariatric surgery is not indicated in patients who are overweight or are only moderately obese. Therefore, there needs to be a systematic approach that combines OSA therapies, including diet/weight loss, physical activity and CPAP in overweight or obese individuals, especially those with other comorbidities.

An important point raised in this context is whether the observed benefits of physical activity might be partly mediated by its impact on BMI. Indeed, disentangling the specific effect of physical activity from BMI reduction remains complex, as these factors are often intertwined. Some evidence suggests that physical activity alone can lead to modest yet significant body weight reductions, independent of dietary or other interventions, as shown in a recent meta-analysis by *Jayedi* et al.^79^ Conversely, *Mendelson* et al. found no significant association between physical activity and BMI changes in patients with OSA.^20^ These discrepancies highlight the need for future studies to control for physical activity when examining BMI-related outcomes in OSA management.

Despite the limited number of RCTs that have evaluated GLP-1 agonists in OSA to date,^26^^,^^80^, ^81^, ^82^ several meta-analyses^25^^,^^36^^,^^61^^,^^83^^,^^84^ have synthesised findings from these studies, suggesting that GLP-1 receptor agonists may offer therapeutic benefits for patients with OSA.

This allowed us to include GLP-1 receptor agonists in our current umbrella meta-analysis. Notably, a phase 3 trial reported that treatment with tirzepatide for one year, alone or combined with positive airway pressure therapy, significantly improved the AHI, body weight, hypoxia, systemic inflammation, systolic BP, and sleep-related QoL compared with placebo. These encouraging findings suggest that GLP-1 receptor agonists may become an important adjunctive therapy for OSA, although further high-quality trials are needed to confirm the clinical benefits of these agents.

Oral surgery and myofunctional therapy alone did not appear to be a suitable treatment option for OSA based on the umbrella review findings. It did not have any significant effect on the AHI, daytime sleepiness, either alone or in combination with an MAD. In a personalised approach to OSA management, there may be some individuals for whom oral surgery represents an appropriate treatment option, but performing repeat polysomnography after surgery would be recommended to determine whether a meaningful reduction in the AHI has been achieved. Studies suggest that myofunctional therapy may reduce OSA severity, especially in mild to moderate cases, by decreasing airway collapsibility.^18^ While promising, this approach generally requires patient adherence over extended periods and is best considered as part of a multimodal approach. More large-scale randomised controlled trials are needed to confirm the effectiveness of myofunctional therapy and determine which patient populations are likely to obtain the greatest benefit. It is a similar situation for HNS—while there may be individuals for whom this is appropriate, it is not currently a widely applicable and effective treatment option for OSA.

In addition to providing a summary of the best published evidence for the impact of currently available treatments on a variety of effectiveness measures based on RCT data, this umbrella review also identified several important knowledge gaps regarding treatment for OSA. While there was a large amount of data relating to CPAP (and to a lesser extent MADs) and the comparative effectiveness of these two therapies, there was a lack of information about other treatments for OSA. In particular, there were very few comparative studies of non-CPAP therapies. There was also a lack of long-term randomised intervention studies for any treatment other than CPAP. The impact of switching from one intervention to another has also been poorly studied to date, as has the comparative effectiveness of different treatment combinations. The latter is particularly relevant for personalised care in clinical practice where the best results are likely to be achieved by combining different treatment modalities and lifestyle modifications. While adherence to CPAP has been very well characterised, this important aspect of therapy has been poorly described for other interventions, including MADs, weight loss and physical activity. This means that the potential usefulness and effects of these treatments over the long term is unknown. Another significant data gap relates to the safety of OSA treatments, apart from pharmacological therapy with wake stimulating agents. This is major limitation of the published RCTs in this field. Finally, although wake stimulating agents were shown to have a positive impact on subjective daytime sleepiness, the effects of these agents on objective sleep study parameters (such as the AHI) is unknown.

A key strength of this study is that it provides an overview of currently available treatments used in patients with OSA to help clinicians decide on the most appropriate options for each individual patient. Furthermore, our findings are based on data from RCTs, which are considered the gold standard study design. We also extracted the results of each RCT from the included meta-analyses and reanalysed data from these meta-analyses to ensure the reliability of the comparisons and findings. In addition, we included a range of therapies used in patients with OSA, which is relatively uncommon in the literature.

There are also some limitations that need to be considered when interpreting our findings. The first relates to the choice to include the meta-analysis with the highest number of included studies for each treatment. This approach may favour larger but lower-quality meta-analyses over smaller, more methodologically rigorous or recent ones, and a high number of included studies does not necessarily reflect the most up-to-date evidence, especially in a rapidly evolving field. Furthermore, meta-analyses with more studies may apply broader or less stringent inclusion criteria, potentially increasing heterogeneity and diluting effect estimates, but our analysis only included meta-analyses of randomised controlled trials). In addition, although numerical size is an objective criterion, relying on this as the primary selection criteria may still introduce subjectivity, especially in cases where two meta-analyses include a similar number of studies but differ in scope or population. Another potential limitation is the absence of a formal peer review of our search strategy by a medical librarian. While such a review is recommended by PRISMA-S and AMSTAR 2 guidelines, our searches were carefully developed by our research team, which included experts in systematic reviews, sleep medicine and clinical trial methodology, and searches were performed across four major databases. This approach substantially mitigates the potential impact of a lack of formal librarian review.

Regarding data analysis, it is important to consider the timing of outcome assessments when interpreting the comparative effectiveness of treatments for OSA. Our analysis primarily included studies with short-to medium-term follow-up, which may explain why oral surgery appeared less effective than MAD in improving the AHI. However, long-term data suggest that the effectiveness of MAD therapy is preserved or may even increase over time, while the durability of surgical outcomes can vary depending on patient-specific factors and the type of procedure performed. Therefore, apparent differences in effectiveness might be influenced by the length of follow-up, highlighting the need for more long-term comparative studies to better assess the sustainability of benefit with these treatment modalities.

Also, there are limitations related to the quality of the selected meta-analyses. First, the majority were critically low in confidence according to AMSTAR 2 and GRADE. Of note, we found many errors and discrepancies between original RCT and extracted data from meta-analyses (notably confusion between SD and SEM [standard error of the mean], interquartile range and 95% CI). Secondly, the definition of the control groups may have been inconsistent between meta-analyses that included the same RCTs. For example, in some studies, the control was reported as “inactive”, while in others, it was identified as “sleep hygiene” or “diet”. To address this, we adhered to the control group definitions from the original RCTs rather than those provided by the meta-analyses. Thirdly, study quality and sample sizes varied substantially between the different interventions assessed, contributing to difficulty in interpreting some findings. For example, while oral surgery did not improve the AHI, it was associated with the largest decrease in BP (although this is based on data from only 65 patients in 1 study). Fourth, there were no meta-analyses of RCTs of new pharmacological interventions and existing RCTs have included a small number of patients with short-term follow-up, which is why these were not included in the umbrella review. Furthermore, while we only included recent meta-analyses some of them may not have included the latest data and could benefit from being updated.^85^ A general limitation of umbrella reviews is the time lag between the publication of impactful individual RCTs and their incorporation into meta-analyses. As such, some recent high-quality RCTs may not yet be reflected in our synthesis. Finally, while RCTs represent the most robust level of scientific evidence, these often include highly selected populations that may not adequately reflect the patients with OSA being treated in clinical practice.^86^^,^^87^ As a result, it is possible that a similar umbrella review of observational study data could yield different findings. We therefore suggest that using the same umbrella review strategy with high-quality observational study data could provide complementary real-world evidence to help inform the personalised management of OSA. One approach that could facilitate better understanding is network meta-analysis, which would allow estimation of the comparative effectiveness of different treatments that have not yet been directly compared in clinical trials, and of different treatment combinations. Network meta-analysis can also help with exploring which treatments are more effective for specific subgroups of patients, providing personalised treatment guidance, and giving clinicians greater insight into which treatments used in patients with OSA may offer the best balance between efficacy, side effects, and patient adherence.

The results of this umbrella review suggest that CPAP generally remains the most effective treatment option for patients with OSA. However, there are also a number of other interventions that have beneficial impacts on different aspects of OSA, including symptoms and QoL. To implement a personalised approach to OSA management it is likely that the best results will be achieved with a combination of interventions. However, long-term data are lacking for the majority of combinations, and comparisons between different interventions and intervention combinations are scarce. Emerging therapies are under investigation, including pharmacological agents based on individual pathophysiological traits. Of these, GLP-1 agonists have recently shown the potential to reduce OSA severity. However, evidence remains limited and further research is needed to establish the role of GLP-1 agonists for the management of OSA. More broadly, there is a clear need for high-quality trials to evaluate the long-term efficacy, safety, and cost-effectiveness of GLP-1 agonists, both as monotherapy and in combination with other treatments for patients with OSA. Additional research is needed to better understand the long-term clinical and economic implications of the full spectrum of OSA therapies.

## Contributors

JLP had the idea for the article. CF and RBM performed the literature search and the acquisition of data. CK contributed to the analysis of the data.

CK and JLP, RBM, CF, SB, MFJ verified the underlying study data. All authors had access to the data study.

CF, RBM and CK drafted the article, JLP wrote the final version of the article.

MJF, SB, RT, and MD critically revised the manuscript for important intellectual content.

All authors had full access to the study documents. All authors contributed to the manuscript revision, read and approved the submitted version.

JLP is the guarantor (the contributor who accepts full responsibility for the finished article, had access to any data, and controlled the decision to publish).

The corresponding author attests that all listed authors meet authorship criteria and that no others meeting the criteria have been omitted.

## Data sharing statement

No new data were generated during this study. All data extracted for the umbrella review are made available with publication in the Open Science Framework (https://osf.io/2jvsx). Any further details are contained in the original publications.

## Declaration of interests

JLP has received grants or contracts from the National Research Agency, and lecture fees and travel grants from Resmed, SEFAM and Bioprojet. SB has received a grant from INNOVADOM (Agiradom), payment or honoraria for lectures, presentations, speakers’ bureaus, manuscript writing or educational events from Vitalaire, Bioprojet, Resmed and Jazz Pharmaceuticals, and support for attending meetings and/or travel from Agiradom, Vitalaire and Bioprojet. RT has received support grants or contracts from Bioprojet (paid directly to his institute), consulting fees (paid to his institute) from Bioprojet, Jazz Pharmaceuticals, Resmed and Idorsia, payment or honoraria for lectures, presentations, speakers bureaus, manuscript writing or educational events from Jazz Pharmaceuticals, Bioprojet, Resmed, Idorsia, Inspire and Elivie, support for attending meetings and/or travel from Agiradom, and has received fees for participation in a Data Safety Monitoring Board or Advisory Board for Bioprojet and Naval (Resmed) (paid to his institute). CF, RBM, CK, MJF, and MD have no conflicts of interest to declare.
