# Supplementary material for: Effect of sleep apnoea interventions on multiple health outcomes: an umbrella review of meta-analyses of randomised controlled trials
Source: eClinicalMedicine. 2025 Oct 8;89:103529. doi: 10.1016/j.eclinm.2025.103529 (PMC12547021; doi:10.1016/j.eclinm.2025.103529)
Supplement: Appendix Tables [file mmc1.pdf]

SUPPLEMENTARY APPENDIX

Effect of sleep apnoea interventions on multiple health outcomes: an umbrella review of meta-analyses of randomised controlled trials

Camille Figard, MD[1]*, Raoua Ben Messaoud, PhD[2]*, Sébastien Baillieul, MD[1,2], Marie Joyeux-Faure, PharmD[1,2], Marie Destors, MD[2], Renaud Tamisier, MD[1,2], Charles Khouri, PharmD[2,3§], Jean-Louis Pépin, MD[1,2§]

[1]EFCR Laboratory, Thorax and Vessels Division, Grenoble Alpes University Hospital, Grenoble, France

[2]Univ. Grenoble Alpes, HP2 Laboratory, Inserm U1300, Grenoble, France

[3]Univ. Grenoble Alpes, Regional Pharmacovigilance Center & Clinical Pharmacology Unit, Grenoble Alpes University Hospital, Grenoble, France

Appendix Table A. Literature search strategy in PubMed, Embase, Web of Science, Cochrane Database of Systematic Reviews

1: "Obstructive sleep apnoea" OR "OSA" OR "Sleep apnoea"[Mesh terms]
2: "Treatments» OR "Therapy"
3: "meta-analysis" [Title/Abstract] OR “systematic review” [Title/Abstract] OR “systematic overview” [Title/Abstract] OR (meta analy*) OR (metaanaly*) OR “systematic AND (review* or overview” AND “Review”
4: "Stimulants" OR "Solriamfetol" OR "Pitolisant" OR "Modafinil"
5: Mandibular advancement device OR MAD or Oral appliance
6; Continuous Positive Airway Pressure" OR "CPAP"
7: "Weight loss" OR "Diet" OR "Low caloric intake"
8: "Physical activity" OR "Exercise"
9: "Bariatric surgery"
10: "GLP-1" OR "Glucose ligand protein" OR "Liraglutide" OR "Tirzepatide"
11: "myofunctional therapy" OR “Oropharyngeal therapy" OR "muscle respiratory training" OR "Orofacial therapy"
10: "1 AND 2 AND 3 AND 4"OR"1 AND 2 AND 3 AND 5"OR"1 AND 2 AND 3 AND 6"OR"1 AND 2 AND 3 AND 7"OR"1 AND 2 AND 3 AND 8"OR 1 AND 2 AND 3 AND 9" OR " 1 AND 2 AND 3 AND 10" AND "1 AND 2 AND 3 AND 11"

Meta-analyses selection and inclusion

- **Methodological scope of umbrella reviews:** Umbrella reviews are designed to synthesise cumulative evidence, rather than to detect the impact of the most recent single trial. Umbrella reviews have been particularly useful in topic with many overlapping meta-analyses, allowing readers to see the “big picture” without duplicating lower-level syntheses”.[1] Their strength lies in overviewing trends, assessing consistency across studies, and evaluating certainty of evidence (using GRADE), which cannot be reliably done with individual randomised controlled trials (RCTs) alone.
- **Literature search:** Our search covers the period from 1 January 2017 to 5 July 2025. The rationale for starting the search in 2017 was to focus on the latest and most clinically relevant evidence. However, it is important to note that meta-analyses published from 2017 onwards typically include RCTs that were conducted and published before 2017.

Therefore, earlier high-quality studies are still captured through their inclusion in these meta-analyses. We also selected the meta-analyses based on the number of RCTs they included, many of which predate 2017. Despite this approach, we often found dozens of meta-analyses addressing the same outcome-treatment pair, indicating a certain level of redundancy in the recent literature. In addition, for CPAP, we manually reviewed earlier literature to ensure that no more relevant or higher-quality meta-analyses published before 2017 had been missed. This check confirmed that our selection strategy did not overlook any better or more comprehensive meta-analyses from earlier years. The search strategy did not undergo formal peer review by a medical librarian, which is a potential limitation of our study. However, although such a review is recommended by the PRISMA-S guidelines and considered a critical component of the AMSTAR 2 methodology, our search was developed and refined collaboratively by our research team, which includes experts in systematic reviews, sleep medicine, and clinical trial methodology. We conducted searches across four major databases (PubMed, Embase, Cochrane Library, and Web of Science), applied a combination of MeSH terms and free-text keywords (Appendix Table A) and followed PRISMA and PRISMA-S guidance. Reference lists of included studies and relevant reviews were also screened to ensure comprehensiveness. We believe that the combination of a thorough multi-database strategy and high levels of expertise in the study team substantially mitigate the impact of no formal librarian review.

- **Clarification on meta-analysis selection:** Our approach involved selecting the meta-analysis with the highest number of RCTs for each intervention-outcome pair, maximising the likelihood that the most comprehensive and up-to-date evidence base was captured. We acknowledge that the number of RCTs alone does not guarantee methodological superiority. However, we applied this rule systematically and transparently across all outcomes to minimise subjective judgment and avoid selection bias. In instances where multiple meta-analyses included the same number of RCTs, we used the AMSTAR 2 tool to evaluate methodological quality. The meta-analysis with the highest AMSTAR rating was then selected for inclusion. Importantly, this selection process, including AMSTAR scoring, was carried out independently by two authors, with

disagreements resolved through discussion and consensus. This ensured objectivity and strengthened the reliability of our quality assessments.

- **Avoiding redundancy:** Including several meta-analyses covering the same outcome-treatment pair would result in redundant data being counted multiple times. This would lead to artificially narrowed confidence intervals and potentially misleading conclusions. Such redundancy can also obscure the actual contribution of individual studies and exaggerate the precision of effect estimates. Moreover, methodological heterogeneity between the different meta-analyses such as differences in inclusion criteria, effect size calculations, and statistical models make it difficult to harmonise the results in a meaningful and unbiased way. Therefore, combining these meta-analyses would not improve precision or reliability but rather amplify inconsistencies.
- **Reanalysis and standardisation:** To address heterogeneity in reporting and quality among the selected meta-analyses, it is important to note that we extracted and reanalysed RCT-level data whenever possible. This step allowed us to apply consistent quality appraisal (GRADE)[2] and to recalculate standardised effect sizes, irrespective of the original methodology or statistical choices of the individual meta-analysis. As such, the limitations of any single meta-analysis are mitigated by our standardised re-evaluation process.

Appendix Table B. Outcomes and measurements

Outcome	Parameter	Scaling	Scoring
**Efficacy**			
Respiratory events	AHI	Continuous (events/h)	NA
Subjective Sleepiness	ESS	Continuous	0 to 24
Quality of life	PGI	Continuous	NA
	CGI	Continuous	NA
	SF-36 questionnaire	Continuous	0 to 100
	FOSQ	Continuous	5 to 20
	SAQLI	Continuous	2.1 to 6.8
**Safety**	Adverse events (serious/any)	Continuous	NA
**Adherence**	Mean use	Continuous (h/night)	NA

AHI = apnoea-hypopnea index; CGI = Clinical Global Impression scale; ESS = Epworth Sleepiness Scale; FOSQ = Functional Outcomes of Sleep Questionnaire; NA = not applicable; PGI = Patient Global Impression scale; SAQLI = Sleep Apnoea Quality of Life Index; SF-36 = Short-Form 36 questionnaire.

Data transformation and calculation

Data from the included meta-analyses were reported using highly heterogeneous formats and statistical parameters, which were not directly comparable across studies. For continuous endpoints, Mean (standard deviation [SD]) was considered the standard and most consistently reported format. When results were presented in alternative statistical forms, predefined transformations were applied to ensure comparability[3-6]:

- Conversion of medians and interquartile ranges (or ranges) to mean (SD) was performed using approximation methods assuming a normal distribution, following the approaches described.
- Conversion of standard error (SE) values to SD values using the formula: $SD = SE \times \sqrt{n}$

Whenever data were missing, we attempted to contact the corresponding authors to obtain access to the raw data.

When studies reported only odds ratio (OR) values, these were converted into standardised mean differences (SMDs)[7] using the approximation: $SMD = OR \times \sqrt{3} / \pi$

Alternatively, when converting from an OR to an effect size (ES: SMD), the following formulas were used: ES = log(OR) / 0.551.

Pooling data and multiplicity of scales

Quality of life (QoL) was assessed using a variety of validated patient-reported outcome measures (PROMs) across the included studies, such as the Short Form-36 (SF-36), EuroQol 5-dimension (EQ-5D), Functional Outcomes of Sleep Questionnaire (FOSQ), and others. These tools capture different aspects of health-related QoL but are not directly comparable due to differences in scoring methods, scale ranges, and conceptual domains. When pooling results from studies that assess QoL using different instruments, we use the SMD as the primary effect size metric to express all treatment effects on a common, unitless scale. This facilitates comparison and pooling across heterogeneous measures (such as SF-36, EQ-5D, Sleep Apnea Quality of Life (SAQLI), FOSQ). However, the SMD is not intuitive for clinicians and policymakers because it lacks a meaningful scale. To enhance interpretability of our meta-analysis, we retransformed the pooled SMDs back into the SF-36 metric, which is a well-established and widely used QoL measure. Due to its ubiquity, interpretability, and clinical utility, the SF-36 was the most appropriate candidate for this purpose. To do this, we multiplied the SMDs by 8.05, which corresponds to the baseline standard deviation of the SF-36 total score in the largest included RCT using that scale. This approach is recommended in the Cochrane Handbook[6] and other methodological references[2] because it anchors the effect size in a clinically meaningful scale.

Appendix Table C. Excluded studies after outcome-intervention assessment based on the randomised clinical trial and AMSTAR-2

Author/Year	Intervention	Inactive	nRCT or AMSAR	Outcomes	Exclusion/Inclusion	Reason of exclusion
Gao et al. 2025[8]	PT	Inactive	6	AHI	Included	nRCT/Outcome
Srijithesh et al. 2019[9]			4		Excluded	
Gao et al. 2025[8]	PT	CPAP	6	AHI	Included	nRCT/Outcome
Gao et al. 2019[10]			4		Excluded	
Gao et al. 2025[8]	PT	CPAP	3	ESS	Included	nRCT/Outcome
Srijithesh et al. 2019[9]			1		Excluded	
Gao et al. 2019[10]			3		Excluded	
Gao et al. 2025[8]	PT	CPAP	4	QoL	Included	
Gao et al. 2025[8]	PT	MAD	5	AHI	Included	nRCT/Outcome
Mohamed et al. 2024[11]			4		Excluded	
Gao et al. 2025	PT	MAD	2	Safety	Included	
Mohamed et al. 2024[11]	PT	MAD	3	ESS	Included	
Mohamed et al. 2024[11]	PT	MAD	3	QoL	Included	
Mohamed et al. 2024[11]	PT	MAD	2	Adherence	Included	
Martínez Revuelta et al. 2024[12]	PA	Inactive	13	AHI	Included	nRCT/Outcome
Lins-Filho et al. 2021[13]			12		Excluded	
Mendelson et al. 2018[14]			6		Excluded	
Edwards et al. 2019[15]			4		Excluded	
Peng et al. 2022[16]			9		Excluded	
Lins-Filho et al. 2020[17]	PA	Inactive	4	QoL	Included	
Mendelson et al. 2018[14]	PA	Inactive	4	ESS	Excluded	nRCT/Outcome
Franks et al. 2022[18]			1		Excluded	
Tang et al. 2024[19]			6		Included	
Peng et al. 2022[16]			4		Excluded	
Edwards et al. 2019[15]	WL (diet)	Inactive	4	AHI	Excluded	nRCT/Outcome
Carneiro-Barrera et al. 2019[20]			6		Included	
Carneiro-Barrera et al. 2019[20]	WL (diet)	Inactive	3	ESS	Included	
Edwards et al. 2019[15]	WL (diet) +PA	Inactive	2	AHI	Included	
Carneiro-Barrera et al. 2019[20]	WL (diet) + CPAP	CPAP	1	AHI	Included	

Kovacs et al. 2022[21]	WL (diet) + CPAP	CPAP	4	SBP, DBP	Included	
Kovacs et al. 2022[21]	WL (diet) + CPAP	WL (Diet)	4	SBP, DBP	Included	
Gao et al. 2019[10]	PA	MAD	1	AHI	Included	
Gao et al. 2019[10]	PA	MAD	1	ESS	Included	
Lins-Filho et al. 2021[13]	PA	WL (Diet)	2	AHI	Included	
Martínez Revuelta et al. 2024[12]	PA+ WL (diet)	WL (Diet)	1	AHI	Included	
Tang et al. 2024[19]	OMT	Inactive	12	AHI	Included	nRCT/Outcome
Zhang et al. 2022[22]			2		Excluded	
Lin et al. 2020[23]			2		Excluded	
Dar et al. 2022[24]			4		Excluded	
Hsu et al. 2020[25]			6		Excluded	
Cavalcante-Leao et al. 2021[26]			4		Excluded	
Tang et al. 2024[19]	OMT	Inactive	9	ESS	Included	nRCT/Outcome
Zhang et al. 2022[22]			4		Excluded	
Lin et al. 2020[23]			2		Excluded	
Dar et al. 2022[24]			4		Excluded	
Hsu et al. 2020[25]			5		Excluded	
Cavalcante-Leao et al. 2021[26]			4		Excluded	
Silva de Sousa et al. 2023[27]	OMT	Inactive	4	SBP, DBP	Included	
Rueda et al. 2020[28]	OMT	CPAP	1	AHI	Included	
Rueda et al. 2020[28]	OMT	CPAP	1	ESS	Included	
Ferreira et al. 2025	CPAP+OMT	CPAP	2	AHI	Included	nRCT/Outcome
Rueda et al. 2020[28]			1		Exluded	
Rueda et al. 2020[28]	CPAP+OMT	CPAP	1	ESS	Included	
Rueda et al. 2020[28]	OMT	PA	1	AHI	Included	
Rueda et al. 2020[28]	OMT	PA	1	ESS	Included	
Gao et al. 2019[10]	CPAP	Inactive	23	AHI	Included	
Li et al. 2022[29]	CPAP	Inactive	41	ESS	Included	nRCT/Outcome
Wang et al. 2020[30]			7		Excluded	
Franks et al. 2022[18]			8		Excluded	

Labarca et al. 2020[31]			4		Excluded	
Li et al. 2023[32]			14		Excluded	
Khan et al. 2018[33]			9		Excluded	
Zhu et al. 2018[34]			2		Excluded	
Yan et al. 2018[35]			13		Excluded	
Patil et al. 2019[36]	CPAP	Inactive	17	QoL	Included	nRCT/Outcome
Timkova et al. 2020[37]			1		Excluded	
Labarca et al. 2020[31]			4		Excluded	
Zheng et al. 2019[38]			14		Excluded	
Brill et al. 2017[39]	CPAP	Inactive	8	Adherence	Included	
Lv et al. 2024[40]	CPAP	Inactive	28, 24	SBP, DBP	Included	nRCT/Outcome
Shang et al. 2021[41]			4		Excluded	
Shang et al. 2022[42]			4		Excluded	
Liu et al. 2024[43]			4		Excluded	
Labarca et al. 2021[44]			3		Excluded	
Lei et al. 2017[45]			6.5		Excluded	
Feng et al. 2023[46]			6		Excluded	
Sun et al. 2024[47]			1		Excluded	
Zhang et al. 2019[38]	CPAP	MAD	13	AHI	Included	nRCT/Outcome
Li et al. 2020[48]			12		Excluded	
Zhang et al. 2019[38]	CPAP	MAD	10	ESS	Included	nRCT/Outcome
Li et al. 2020[48]			7		Excluded	
Schwartz et al. 2018[49]	CPAP	MAD	6	QoL	Included	
Schwartz et al. 2018[49]	CPAP	MAD	6	Adherence	Included	
Gao et al. 2019[10]	MAD	Inactive	12	AHI	Included	nRCT/Outcome
Kuhn et al. 2017[50]			9		Excluded	
Yu M et al. 2023[51]			6		Excluded	
Guo et al. 2024[52]			6		Excluded	
Vila-Nova et al. 2022[53]			1		Excluded	
Gao et al. 2019[10]	MAD	Inactive	9	ESS	Included	nRCT/Outcome
Yu et al. 2023[51]			6		Excluded	
De Vries et al. 2017	MAD	Inactive	5	SBP, DBP	Included	nRCT/Outcome

Yu et al. 2023[51]			1		Excluded	
Rangarajan, et al. 2022[54]	MAD	Inactive	5	QoL	Included	nRCT/Outcome
Yu et al. 2023[51]			4		Excluded	
Vimal et al. 2022[55]	MAD	Inactive	3	Adherence	Included	
Belanche Monterde et al. 2025[56]	MAD	Inactive	8	Day/ night DBP, SBP	Included	
Kang et al. 2022[57]	HNS	Inactive	1	SBP, DBP	Included	
Wollny et al. 2024[58]	HNS	Inactive	2	Safety (any/serious TEAE)	Included	
Alrubasy et al. 2024[59]	HNS	Inactive	3	AHI	Included	nRCT/Outcome
Ratneswaran et al. 2021[60]			1		Excluded	
Alrubasy et al. 2024[59]	HNS	Inactive	10	ESS	Included	AMSTAR
Braun et al. 2023[61]			7	ESS	Excluded	
Altobaishat et al. 2025[62]	GLP-1	Placebo	12	Safety (any/serious TEAE)	Included	AMSTAR
Kow et al. 2025[63]	GLP-1		10		Excluded	
Li et al. 2025[64]	GLP-1	Placebo	9	AHI	Included	AMSTAR
Dutta et al. 2025[65]	GLP-1	GLP-1	7	AHI	Excluded	
Li et al. 2025[64]	GLP-1	Placebo	2	SBP, DBP	Included	
Yang et al. 2025[66]	GLP-1	PLacebo	4	AHI	Included	
Li et al. 2025[64]	GLP-1 (liraglutide)	Placebo	1	SBP, DBP	Included	
Altobaishat et al. 2025[62]	GLP-1 + CPAP	CPAP	1	SBP, DBP	Included	
Altobaishat et al. 2025[62]	GLP-1 (tirzepatide)+ CPAP	CPAP	1	Safety (any/serious TEAE)	Included	
Li et al. 2025[64]	GLP-1 (liraglutide)+ CPAP	CPAP	2	SBP, DBP	Included	

Yang et al. 2025[66]	GLP-1 (liraglutide)+ CPAP	CPAP	1	AHI	Included	
Bardóczi et al. 2025[67]	GLP-1	CPAP	8	AHI	Excluded	AMSTAR
John et al. 2018[68]	Oral surgery	Inactive	1	AHI	Excluded	nRCT/Outcome
Gao et al. 2019[10]			6		Included	
Camacho et al. 2017[69]			2		Excluded	
Lechien et al. 2021[70]			1		Excluded	
Saenwandee et al. 2022[71]			2		Excluded	
Schoustra et al. 2022[72]			1		Excluded	
Gao et al. 2019[10]	Oral surgery	Inactive	6	ESS	Included	nRCT/Outcome
Lechien et al. 2021[70]			1		Excluded	
Saenwandee et al. 2022[71]			1		Excluded	
Schoustra et al. 2022[72]			1		Excluded	
Kang et al. 2022[57]	Oral surgery	Inactive	1	BP	Included	
Zhou et al. 2021[73]	Oral surgery	CPAP	1	AHI	Included	
Gao et al. 2019[10]	Oral surgery	CPAP	1	ESS	Included	
He et al. 2018[74]	Oral surgery	MAD	1	AHI	Included	
Gao et al. 2019[10]	Oral surgery + MAD	Oral surgery	1	AHI	Included	
Zhang et al. 2019[75]	BS	Inactive	1	AHI	Included	
Locke et al. 2024[76]	BS	CPAP	1	AHI	Included	
Kou et al. 2022[77]	BS	CPAP	1	SBP, DBP	Included	
Wong et al. 2018[78]	BS	Diet	2	AHI	Included	
Malhotra et al. 2024[79]	GLP-1	Placebo			Included	
Pépin et al. 2024[80]	Pitolisant	Placebo	13	ESS	Included	AMSTAR
Wang J et al. 2021[81]			12		Excluded	
Pitre et al. 2023[82]			12		Excluded	
Pépin et al. 2024[80]	Pitolisant	Placebo	13	QoL	Included	nRCT/Outcome
Wang J et al. 2021[81]			12		Excluded	
Pépin et al. 2024[80]	Pitolisant	Placebo	13	Safety	Included	nRCT/Outcome
Wang J et al. 2021[81]			12		Excluded	

Pépin et al. 2024[80]	Solriamfetol	Placebo	6	QoL	Included	nRCT/Outcome
Wang J et al. 2021[83]			2		Excluded	
Ronnebaum et al. 2021[84]			1		Excluded	
Pépin et al. 2024[80]	Solriamfetol	Placebo	3	Safety (any serious/most frequent TEAE)	Included	nRCT/Outcome
Subedi et al. 2020[85]			1		Excluded	
Wang J et al. 2021[83]			2		Excluded	
Ronnebaum et al. 2021[84]			1		Excluded	
Pépin et al. 2024[80]	Solriamfetol	Placebo	20	ESS	Included	nRCT/Outcome
Ronnebaum et al. 2021[84]			1		Excluded	
Neshat et al. 2024[86]			16		Included	
Wang J et al. 2021[83]			3		Excluded	
Subedi et al. 2020[45]			3		Excluded	
Pitre et al. 2023[82]			1		Excluded	
Pépin et al. 2024[80]	Modafinil	Placebo	9	QoL	Included	nRCT/Outcome
Ronnebaum et al. 2021[84]			5		Excluded	
Pépin et al. 2024[80]	Modafinil	Placebo	10	Safety (any serious/most frequent TEAE)	Included	nRCT/Outcome
Ronnebaum et al. 2021[84]			5		Excluded	
Pépin et al. 2024[80]	Modafinil	Placebo	12	ESS	Included	AMSTAR+ nRCT/Outcome
Ronnebaum et al. 2021[84]			5		Excluded	
Pitre et al. 2023[82]			12		Excluded	

AHI = apnoea-hypopnoea index; BP = blood pressure; CPAP = continuous positive airway pressure; CV = cardiovascular; ESS = Epworth Sleepiness Scale; HNS = hypoglossal nerve stimulation; MAD = mandibular advancement device; nRCT = number of randomised clinical trials; PA = physical activity; QoL = quality of life; TEAE = treatment-emergent adverse effects; WL = weight loss.

Appendix Table D. AMSTAR 2 Evaluation method, critical domains, and interpretation

AMSTAR 2

1. Did the research questions and inclusion criteria for the review include the components of PICO?

For Yes:	Optional (recommended)	
☐ Population<br>☐ Intervention<br>☐ Comparator group<br>☐ Outcome	☐ Timeframe for follow-up	☐ Yes<br>☐ No

2. Did the report of the review contain an explicit statement that the review methods were established prior to the conduct of the review and did the report justify any significant deviations from the protocol?

For Partial Yes:	For Yes:	
The authors state that they had a written protocol or guide that included ALL the following:<br>☐ review question(s)<br>☐ a search strategy<br>☐ inclusion/exclusion criteria<br>☐ a risk of bias assessment	As for partial yes, plus the protocol should be registered and should also have specified:<br>☐ a meta-analysis/synthesis plan, if appropriate, *and*<br>☐ a plan for investigating causes of heterogeneity<br>☐ justification for any deviations from the protocol	☐ Yes<br>☐ Partial Yes<br>☐ No

3. Did the review authors explain their selection of the study designs for inclusion in the review?

For Yes, the review should satisfy ONE of the following:	
☐ *Explanation for* including only RCTs<br>☐ OR *Explanation for* including only NRSI<br>☐ OR *Explanation for* including both RCTs and NRSI	☐ Yes<br>☐ No

4. Did the review authors use a comprehensive literature search strategy?

For Partial Yes (all the following):	For Yes, should also have (all the following):	
☐ searched at least 2 databases (relevant to research question)<br>☐ provided key word and/or search strategy<br>☐ justified publication restrictions (eg, language)	☐ searched the reference lists/bibliographies of included studies<br>☐ searched trial/study registries<br>☐ included/consulted content experts in the field<br>☐ where relevant, searched for grey literature<br>☐ conducted search within 24 months of completion of the review	☐ Yes<br>☐ Partial Yes<br>☐ No

5. Did the review authors perform study selection in duplicate?

For Yes, either ONE of the following:	
☐ at least two reviewers independently agreed on selection of eligible studies and achieved consensus on which studies to include<br>☐ OR two reviewers selected a sample of eligible studies and achieved good agreement (at least 80 per cent), with the remainder selected by one reviewer	☐ Yes<br>☐ No

6. Did the review authors perform data extraction in duplicate?

For Yes, either ONE of the following:	
☐ at least two reviewers achieved consensus on which data to extract	☐ Yes

from included studies<br>☐ OR two reviewers extracted data from a sample of eligible studies <u>and</u> achieved good agreement (at least 80 per cent), with the remainder extracted by one reviewer		☐ No

7. Did the review authors provide a list of excluded studies and justify the exclusions?

For Partial Yes:	For Yes, must also have:	
☐ provided a list of all potentially relevant studies that were read in full text form but excluded from the review	☐ Justified the exclusion from the review of each potentially relevant study	☐ Yes<br>☐ Partial Yes<br>☐ No

8. Did the review authors describe the included studies in adequate detail?

For Partial Yes (ALL the following):	For Yes, should also have ALL the following:	
☐ described populations<br>☐ described interventions<br>☐ described comparators<br>☐ described outcomes<br>☐ described research designs	☐ described population in detail<br>☐ described intervention and comparator in detail (including doses where relevant)<br>☐ described study's setting<br>☐ timeframe for follow-up	☐ Yes<br>☐ Partial Yes<br>☐ No

9. Did the review authors use a satisfactory technique for assessing the risk of bias (RoB) in individual studies that were included in the review?

**RCTs**<br>For Partial Yes, must have assessed RoB from<br>☐ unconcealed allocation, *and*<br>☐ lack of blinding of patients and assessors when assessing outcomes (unnecessary for objective outcomes such as all cause mortality)	For Yes, must also have assessed RoB from:<br>☐ allocation sequence that was not truly random, *and*<br>☐ selection of the reported result from among multiple measurements or analyses of a specified outcome	☐ Yes<br>☐ Partial Yes<br>☐ No<br>☐ Includes only NRSI
**NRSI**<br>For Partial Yes, must have assessed RoB:<br>☐ from confounding, *and*<br>☐ from selection bias	For Yes, must also have assessed RoB:<br>☐ methods used to ascertain exposures and outcomes, *and*<br>☐ selection of the reported result from among multiple measurements or analyses of a specified outcome	☐ Yes<br>☐ Partial Yes<br>☐ No<br>☐ Includes only RCTs

10. Did the review authors report on the sources of funding for the studies included in the review?

For Yes<br>☐ Must have reported on the sources of funding for individual studies included in the review. Note: Reporting that the reviewers looked for this information but it was not reported by study authors also qualifies	☐ Yes<br>☐ No

11. If meta-analysis was performed did the review authors use appropriate methods for statistical combination of results?

**RCTs**<br>For Yes:<br>☐ The authors justified combining the data in a meta-analysis<br>☐ AND they used an appropriate weighted technique to combine study results and adjusted for heterogeneity if present	☐ Yes<br>☐ No<br>☐ No meta-analysis

☐ AND investigated the causes of any heterogeneity	conducted
**For NRSI**<br>For Yes:<br>☐ The authors justified combining the data in a meta-analysis<br>☐ AND they used an appropriate weighted technique to combine study results, adjusting for heterogeneity if present<br>☐ AND they statistically combined effect estimates from NRSI that were adjusted for confounding, rather than combining raw data, or justified combining raw data when adjusted effect estimates were not available<br>☐ AND they reported separate summary estimates for RCTs and NRSI separately when both were included in the review	☐ Yes<br>☐ No<br>☐ No meta-analysis conducted

12. If meta-analysis was performed, did the review authors assess the potential impact of RoB in individual studies on the results of the meta-analysis or other evidence synthesis?

For Yes:<br>☐ included only low risk of bias RCTs<br>☐ OR, if the pooled estimate was based on RCTs and/or NRSI at variable RoB, the authors performed analyses to investigate possible impact of RoB on summary estimates of effect	☐ Yes<br>☐ No<br>☐ No meta-analysis conducted

13. Did the review authors account for RoB in individual studies when interpreting/discussing the results of the review?

For Yes:<br>☐ included only low risk of bias RCTs<br>☐ OR, if RCTs with moderate or high RoB, or NRSI were included the review provided a discussion of the likely impact of RoB on the results	☐ Yes<br>☐ No

14. Did the review authors provide a satisfactory explanation for, and discussion of, any heterogeneity observed in the results of the review?

For Yes:<br>☐ There was no significant heterogeneity in the results<br>☐ OR if heterogeneity was present the authors performed an investigation of sources of any heterogeneity in the results and discussed the impact of this on the results of the review	☐ Yes<br>☐ No

15. If they performed quantitative synthesis did the review authors carry out an adequate investigation of publication bias (small study bias) and discuss its likely impact on the results of the review?

For Yes:<br>☐ performed graphical or statistical tests for publication bias and discussed the likelihood and magnitude of impact of publication bias	☐ Yes<br>☐ No<br>☐ No meta-analysis conducted

16. Did the review authors report any potential sources of conflict of interest, including any funding they received for conducting the review?

For Yes:<br>☐ The authors reported no competing interests OR<br>☐ The authors described their funding sources and how they managed potential conflicts of interest	☐ Yes<br>☐ No

AMSTAR 2 critical domains

- Protocol registered before commencement of the review **(item 2)**
- Adequacy of the literature search **(item 4)**
- Justification for excluding individual studies **(item 7)**
- Risk of bias from individual studies being included in the review **(item 9)**

- Appropriateness of meta-analytical methods **(item 11)**
- Consideration of risk of bias when interpreting the results of the review **(item 13)**
- Assessment of presence and likely impact of publication bias **(item 15)**

AMSTAR 2 interpretation

High

- No or one non-critical weakness: the systematic review provides an accurate and comprehensive summary of the results of the available studies that address the question of interest

Moderate

- More than one non-critical weakness: the systematic review has more than one weakness but no critical flaws. It may provide an accurate summary of the results of the available studies that were included in the review

Low

- One critical flaw with or without non-critical weaknesses: the review has a critical flaw and may not provide an accurate and comprehensive summary of the available studies that address the question of interest

Critically low

- More than one critical flaw with or without non-critical weaknesses: the review has more than one critical flaw and should not be relied on to provide an accurate and comprehensive summary of the available studies

Appendix Table E. Study quality evaluation using the Assessment of Multiple Systematic Reviews 2 (AMSTAR 2).

Metaanalysis	Intervention	Control	Amstar items																	Analysis
			Item 1	Item 2*	Item 3	Item 4*	Item 5	Item 6	Item 7*	Item 8	Item 9*	Item 10	Item 11*	Item 12	Item 13*	Item 14	Item 15*	Item 16	Score	
Alrubasy et al. 2025[59]	HNS	Inactive	1	1	0	0	1	1	0	1	1	0	1	0	0	1	1	1	10	Critically low
Altobaishat et al. 2025[62]	GLP-1	Inactive	1	1	0	0	1	1	0	1	1	0	1	0	0	1	1	1	10	Critically low
Li et al. 2025[64]	GLP-1	Inactive	1	0	0	0	1	1	0	1	1	0	1	1	0	1	1	0	9	Critically low
Yang et al 2025[66]	GLP-1	Inactive	1	1	0	0	0	1	0	1	1	0	1	1	1	1	1	1	11	Critically low
Locke et al. 2024[76]	OMT	Inactive	1	1	0	0	1	1	0	1	1	0	1	1	1	1	1	1	12	Critically low
Revuelta et al. 2024[12]	PA<br>WL (Diet)	Inactive<br>WL (Diet)	1	1	0	0	1	1	0	1	1	0	1	1	1	1	1	1	12	Critically low
Lv et al 2024[40]	CPAP	Inactive	1	1	0	0	1	1	0	1	1	0	1	1	1	1	1	1	12	Critically low
Rueda et al 2020[28]	OMT	Inactive	1	1	1	0	1	1	1	1	1	1	1	1	1	1	1	1	15	low
Tang et al. 2024[19]	PA<br>OMT	Inactive	1	1	0	0	1	1	0	1	1	0	1	1	1	1	1	1	12	Critically low
Wollny et al. 2024[58]	HNS	Inactive	1	1	0	0	1	1	0	1	1	0	1	1	1	1	1	1	12	Critically low
De Sousa et al. 2023[27]	OMT	Inactive	1	1	0	0	1	1	0	1	1	0	1	1	1	1	1	1	12	Critically low
Ferreira et al. 2025[87]	OMT<br>CPAP<br>PA	Inactive<br>CPAP	1	1	0	0	1	1	0	1	1	0	1	1	1	1	1	1	12	Critically low
Gao et al. 2025[8]	PT<br>MAD	Inactive<br>PT<br>MAD	1	0	0	1	1	0	1	0	1	0	1	1	1	1	1	1	11	Critically low
Gao et al 2019[10]	CPAP	Inactive	1	0	1	1	1	1	1	1	1	0	0	1	0	0	0	1	9	Critically low
Li et al. 2022[29]	CPAP	Inactive	1	0	1	1	0	0	0	1	1	0	1	0	0	1	1	1	8	Critically low

Patil et al. 2019[36]	CPAP	Inactive	1	0	0	1	1	1	0	0	0	0	0	0	0	1	1	1	6	Critically low
Brill et al. 2017[39]	CPAP	Inactive	1	0	1	1	1	1	0	1	1	0	1	0	0	1	0	1	9	Critically low
Zhang et al. 2019[88]	CPAP	MAD	1	1	1	1	1	0	0	1	1	0	1	0	1	1	1	1	11	moderate
Gao et al 2019[10]	CPAP	MAD	1	0	1	1	1	1	1	1	1	0	0	1	0	0	0	1	9	Critically low
Schwartz et al. 2018[49]	CPAP	MAD	1	1	1	1	1	1	0	1	1	0	1	1	1	1	1	1	13	High
Gao et al 2019[10]	MAD	Inactive	1	0	1	1	1	1	1	1	1	0	0	1	0	0	0	1	9	Critically low
De Vries et al. 2017[89]	MAD	Inactive	1	1	0	1	1	1	0	1	0	0	0	0	0	0	0	1	6	Critically low
Rangarajan et al. 2022[54]	MAD	Inactive	1	1	0	1	1	1	1	1	1	0	1	0	1	0	0	0	10	moderate
Vimal et al. 2022[55]	MAD	Inactive	1	1	1	1	1	1	0	1	1	0	0	1	0	1	0	1	10	Critically low
Gao et al 2019[10]	Positional therapy	CPAP	1	0	1	1	1	1	1	1	1	0	0	1	0	0	0	1	9	Critically low
Lins-Filho et al. 2021[13]	PA	Inactive	1	1	1	1	1	1	0	1	1	0	0	0	0	1	0	1	9	Critically low
Lins-Filho et al. 2020[17]	PA	Inactive	1	1	0	1	1	1	0	1	1	0	0	1	1	0	0	1	9	Low
Gao et al 2019[10]	PA	MAD	1	0	1	1	1	1	1	1	1	0	0	1	0	0	0	1	9	Critically low
Edwards et al. 2019[15]	PA	CPAP	1	1	1	1	1	1	1	1	1	0	1	1	1	1	1	1	14	High
Gao et al 2019[10]	PA	CPAP	1	0	1	1	1	1	1	1	1	0	0	1	0	0	0	1	9	Critically low
Lins-Filho et al. 2021[13]	PA	WL	1	1	1	1	1	1	0	1	1	0	0	0	0	1	0	1	9	Critically low
Carneiro-Barrera et al. 2019[20]	WL	Inactive	1	1	1	1	0	1	0	1	1	0	1	1	0	0	1	1	10	Low
Carneiro-Barrera et al. 2019[20]	WL + CPAP	CPAP	1	1	1	1	0	1	0	1	1	0	1	1	0	0	1	1	10	Low
Kovacs et al. 2022[21]	WL + CPAP	CPAP	1	1	1	1	1	0	0	1	1	0	0	1	0	0	0	1	8	Critically low
Kovacs et al. 2022[21]	WL + CPAP	WL	1	1	1	1	1	0	0	1	1	0	0	1	0	0	0	1	8	Critically low
Wong et al. 2018[78]	Bariatric surgery	WL	1	1	1	1	1	1	0	1	1	0	1	1	1	1	0	1	12	Moderate

Kou et al. 2022[77]	Bariatric surgery	CPAP	1	1	1	1	1	1	0	1	1	0	1	1	1	1	0	1	12	Moderate
Gao et al 2019[10]	Oral surgery	Inactive	1	0	1	1	1	1	1	1	1	0	0	1	0	0	0	1	9	Critically low
Kang et al 2022[57]	Oral surgery	Inactive	1	0	0	1	1	1	0	1	1	0	0	0	0	0	0	1	6	Critically low
Zhou et al. 2021[73]	Oral surgery	CPAP	1	1	0	1	1	1	0	1	1	0	0	0	1	1	0	1	9	Critically low
Gao et al 2019[10]	Oral surgery	CPAP	1	0	1	1	1	1	1	1	1	0	0	1	0	0	0	1	9	Critically low
He et al. 2019[74]	Oral surgery	MAD	1	0	0	1	1	1	0	1	1	0	1	0	0	1	0	1	8	Critically low
Gao et al 2019[10]	Oral surgery + MAD	Oral surgery	1	0	1	1	1	1	1	1	1	0	0	1	0	0	0	1	9	Critically low
Kang et al. 2022[57]	HNS	Inactive	1	0	0	1	1	1	0	1	1	0	0	0	0	0	0	1	6	Critically low
Mohamed et al. 2024[11]	MAD	Positional therapy	1	1	1	1	1	1	0	1	1	0	1	1	1	0	0	1	11	Moderate
Pépin et al. 2024[80]	Modafinil	Inactive	1	1	1	1	1	1	1	1	1	0	1	1	1	1	0	1	13	High
Pépin et al. 2024[80]	Solriamfetol	Inactive	1	1	1	1	1	1	1	1	1	0	1	1	1	1	0	1	13	High
Pépin et al. 2024[80]	Pitolisant	Inactive	1	1	1	1	1	1	1	1	1	0	1	1	1	1	0	1	13	High

AHI = apnoea-hypopnea index; BP = blood pressure; CPAP = continuous positive airway pressure; CV = cardiovascular; DBP = diastolic blood pressure; ESS = Epworth Sleepiness Scale; HNS = hypoglossal nerve stimulation; MACE = major adverse cardiovascular events; MAD = mandibular advancement device; Mod. = moderate; NA = not available; QoL = quality of life; SBP = systolic blood pressure; TEAE = treatment-emergent adverse event; WL = weight loss.

*AMSTAR 2 critical domains.

Appendix Table F. GRADE assessment of included studies[90]

Author	Intervention	Control	Outcomes	N RCT	Metric	Effect size (95% CI)	p_value	I²	P_egger	Downgrade factors					Upgrade factors		Quality
										Risk of bias	Imprecision	Inconsistency	Indirectness	Publication bias	SMD >0.8 or <–0.8	SMD >1.2 or <–1.2	
Gao et al. 2019[10]	CPAP	Inactive	AHI	23	SMD	–1.65 (–1.87;–1.43)	<.0.0001	63.96	0.001	Serious	not serious	not serious	serious concern	serious concern		2	Mod.
Li et al. 2022[29]	CPAP	Inactive	ESS	41	SMD	–0.82 (–1.16;–0.48)	<.0.0001	93.04	0.14	Serious	not serious	very serious concern	not serious	not serious	1		Mod.
Patil et al. 2019[36]	CPAP	Inactive	QoL	20	SMD	0.16(0.11;0.21)	<.0.0001	26.86	0.48	very serious	not serious	not serious	serious concern	not serious	0		Low
Lv et al. 2024[40]	CPAP	Inactive	SBP	28	SMD	–0.12 (–0.18; –0.06)	0	0	0.02	very serious	not serious	not serious	not serious	serious concern	0		Low
Lv et al. 2024[40]	CPAP	Inactive	DBP	27	SMD	–0.18 (–0.24;–0.11)	<.0.0001	4.08	0.03	very serious	not serious	not serious	not serious	serious concern	0		Low
Brill et al. 2017[39]	CPAP	Inactive	Adherence	8	SMD	4.02 (2.85;5.2)	<.0.0001	93.82	NA	very serious	serious concern	very serious concern	not serious	NA		2	Low
Zhang et al. 2019[88]	CPAP	MAD	AHI	13	SMD	–0.83 (–1; –0.67)	<.0.0001	33.15	0.35	very serious	not serious	not serious	not serious	not serious	1		Mod.
Zhang et al. 2019[88]	CPAP	MAD	SBP	4	SMD	0.04 (–0.13;0.21)	0.64	0	NA	very serious	not serious	not serious	not serious	NA	0		Low
Zhang et al. 2019[88]	CPAP	MAD	DBP	4	SMD	0.04 (–0.13;0.22)	0.63	0	NA	very serious	not serious	not serious	not serious	NA	0		Mod.
Gao et al. 2019[10]	CPAP	MAD	ESS	10	SMD	–0.18 (–0.43;0.07)	0.16	68.36	0.44	very serious	not serious	not serious	not serious	not serious	0		Mod.
Schwartz et al. 2018[49]	CPAP	MAD	QoL	6	SMD	0.02 (–0.1;0.14)	0.77	0	NA	very serious	not serious	not serious	not serious	NA	0		Mod.
Schwartz et al. 2018[49]	CPAP	MAD	Adherence	6	SMD	–0.87 (–2.00;–0.27)	0.01	94.31	0.14	very serious	serious concern	very serious concern	not serious	NA	1		Low
Gao et al. 2019[10]	MAD	Inactive	AHI	11	SMD	–0.73 (–0.91;–0.54)	<.0.0001	25.36	0.005	very serious	not serious	not serious	serious concern	serious concern	0		Low
Gao et al. 2019[10]	MAD	Inactive	ESS	8	SMD	–0.34 (–0.53;–0.14)	0	17.52	NA	very serious	not serious	not serious	serious concern	NA	0		Low
De Vries et al. 2017[89]	MAD	Inactive	SBP	5	SMD	–0.11 (–0.29;0.07)	0.002	36.03	NA	very serious	not serious	not serious	not serious	NA	0		Mod.
De Vries et al. 2017[89]	MAD	Inactive	DBP	5	SMD	–0.1 (–0.3;0.1)	0.02	19.32	NA	very serious	not serious	not serious	not serious	NA	0		Mod.
Rangarajan et al. 2022[54]	MAD	Inactive	QoL	3	SMD	–0.3 (–0.80;0.2)	0.24	74.85	NA	serious concerns	serious concern	not serious	not serious	NA	0		Mod.

Author	Intervention	Control	Outcomes	N RCT	Metric	Effect size (95% CI)	p_value	I²	P_egger	Downgrade factors					Upgrade factors		Quality
										Risk of bias	Imprecision	Inconsistency	Indirectness	Publication bias	SMD >0.8 or <–0.8	SMD >1.2 or <–1.2	
Vimal et al. 2022[55]	MAD	Inactive	Adherence	3	SMD	0.43 (0.00;0.86)	0.05	47.09	NA	serious concerns	serious concern	not serious	not serious	NA	0		Mod.
Gao et al. 2025[8]	MAD	Positional	safety (any TEAE)	2	SMD	0.33 (0.03;0.64)	0.03	0	NA	very serious	not serious	not serious	serious concern	NA	1	0	Mod.
Gao et al. 2025[8]	Positional therapy	CPAP	AHI	6	SMD	0.74 (0.52;0.97)	<0.0001	0	NA	very serious	not serious	not serious	serious concern	NA	1	0	Mod.
Gao et al. 2025[8]	Positional therapy	CPAP	ESS	3	SMD	0.2 (–0.09;0.48)	0.18	0	NA	very serious	not serious	not serious	serious concern	NA	0	0	Low
Gao et al. 2025[8]	Positional therapy	CPAP	QoL	4	SMD	-0.08 (–0.34;0.184)	0.56	0	NA	very serious	not serious	not serious	serious concern	NA	0	0	Low
Lins-Filho et al. 2020[17]	Physical activity	Inactive	QoL	4	SMD	1.3 (0.58;2.02)	0	70.89	NA	very serious	serious concern	not serious	not serious	NA	0	2	Mod.
Lins-Filho et al. 2021[17]	Physical activity	Inactive	AHI	13	SMD	–1.13 (–2;–0.66)	0	86.92	0	very serious	not serious	Serious concern	not serious	not serious	0	0	Mod.
Tang et al. 2024[19]	Physical activity	Inactive	ESS	6	SMD	–0.3 (–0.52;–0.09)	0	0	NA	not serious	not serious	not serious	serious concern	NA	0	2	Mod.
Gao et al. 2019[10]	Physical activity	MAD	AHI	1	SMD	0.64 (–0.13;1.41)	0.1	NA	NA	very serious	serious concern	NA	not serious	NA	0	0	Low
Gao et al. 2019[10]	Physical activity	MAD	ESS	1	SMD	0.79 (0.01;1.57)	0.05	NA	NA	very serious	serious concern	NA	not serious	NA	0	0	Low
Edwards et al. 2019[15]	Physical activity	CPAP	AHI	1	SMD	0.11 (–0.48;0.70)	0.71	NA	NA	very serious	serious concern	NA	not serious	NA	0	0	Low
Gao et al. 2019[10]	Physical activity	CPAP	ESS	1	SMD	–0.06 (–0.8;0.69)	0.88	NA	NA	very serious	serious concern	NA	not serious	NA	0	0	Low
Lins–Filho et al. 2021[13]	Physical activity	diet	AHI	2	SMD	–0.7 (–0.1; –0.41)	<0.0001	0	NA	very serious	not serious	not serious	not serious	NA	0	0	Mod.
Martínez Revuelta et al. 2024[12]	Physical activity + Diet	diet	AHI	1	SMD	–0.1 (–0.78; 0.57)	0.76	NA	NA	very serious	Serious concern	NA	not serious	NA	0	0	Mod.
Carneiro-Barrera et al. 2019[20]	Diet	Inactive	AHI	6	SMD	–0.63 (–0.9;–0.36)	<0.0001	49.29	NA	serious concerns	not serious	not serious	not serious	NA	0	0	Mod.
Carneiro-Barrera et al. 2019[20]	Diet	Inactive	ESS	3	SMD	–0.35 (–0.64;–0.06)	0.02	31.18	NA	serious concerns	not serious	not serious	not serious	NA	0	0	Mod.

Author	Intervention	Control	Outcomes	N RCT	Metric	Effect size (95% CI)	p_value	I²	P_egger	Downgrade factors					Upgrade factors		Quality
										Risk of bias	Imprecision	Inconsistency	Indirectness	Publication bias	SMD >0.8 or <–0.8	SMD >1.2 or <–1.2	
Carneiro-Barrera et al. 2019[20]	Diet + CPAP	CPAP	AHI	1	SMD	–0.71 (–1.15;–0.27)	0	NA	NA	serious concerns	not serious	NA	not serious	NA	0	0	Mod.
Kovács et al. 2022[21]	Diet + CPAP	CPAP	SBP	4	SMD	–1.61 (–3.78;0.55)	0.14	97.71	NA	serious concerns	serious concern	very serious concern	not serious	NA	0	2	Mod.
Kovács et al. 2022[21]	Diet + CPAP	CPAP	DBP	4	SMD	–1.87 (–4.58;0.84)	0.17	98.05	NA	serious concerns	very serious	very serious concern	not serious	NA	0	2	Low
Kovács et al. 2022[21]	Diet + CPAP	diet	SBP	4	SMD	–0.21 (–0.6;0.19)	0.31	76.09	NA	serious concerns	not serious	serious concern	not serious	NA	0	0	Mod.
Kovács et al. 2022[21]	Diet + CPAP	diet	DBP	4	SMD	–0.49 (–1.03;0.05)	0.007	86.46	NA	serious concerns	serious concern	serious concern	not serious	NA	0	0	Low
Wong et al. 2018[78]	Bariatric surgery	diet	AHI	2	SMD	–0.5 (–0.89;–0.11)	0.01	0	NA	very serious	not serious	not serious	not serious	NA	0	0	Mod.
Locke et al. 2024[76]	Bariatric surgery	CPAP	AHI	2	SMD	0.14 (–0.41;0.7)	0.60	NA	NA	not serious	Serious concern	NA	not serious	NA	0	0	Mod.
Kou et al. 2022[77]	Bariatric surgery	CPAP	SBP	1	SMD	–0.36 (–1.17;0.44)	0.36	NA	NA	very serious	serious concern	NA	not serious	NA	0	0	Low
Kou et al. 2022[77]	Bariatric surgery	CPAP	DBP	1	SMD	–0.42 (–1.23;0.38)	0.29	NA	NA	very serious	serious concern	NA	not serious	NA	0	0	Low
Gao et al. 2019[10]	Oral surgery	Inactive	AHI	6	SMD	–0.40 (–0.69;–0.11)	0	42.18	NA	very serious	not serious	not serious	serious concern	NA	0	0	Low
Gao et al. 2019[10]	Oral surgery	Inactive	ESS	6	SMD	–0.29 (–0.61;0.03)	0.07	51.18	NA	very serious	not serious	not serious	serious concern	NA	0	0	Low
He et al. 2019[74]	Oral surgery	MAD	AHI	1	SMD	0.62 (0.17;1.08)				very serious	serious concern	NA	not serious	NA		0	Low
Kang et al. 2022[57]	Oral surgery	Inactive	SBP	1	SMD	–0.54 (–1.04;–0.03)	0.04	NA	NA	serious concerns	serious concern	NA	not serious	NA	0	0	Mod.
Kang et al. 2022[57]	Oral surgery	Inactive	DBP	1	SMD	–0.49 (–1.00;0.01)	0.06	NA	NA	serious concerns	not serious	NA	not serious	NA	0	0	Mod.
Zhou et al. 2021[73]	Oral surgery	CPAP	AHI	1	SMD	0.35 (–0.22; 0.93)	0.22	NA	NA	very serious	serious concern	NA	not serious	NA	0	0	Low
Gao et al. 2019[10]	Oral surgery	CPAP	ESS	1	SMD	–0.04 (–0.56;0.48)	0.88	NA	NA	very serious	serious concern	NA	not serious	NA	0	0	Low
He et al. 2019[74]	Oral surgery	MAD	AHI	1	SMD	0.62 (0.17;1.08)	0.01	NA	NA	very serious	serious concern	NA	not serious	NA	0	0	Low
Gao et al. 2019[10]	Oral surgery + MAD	oral surgery	AHI	1	SMD	0.18 (–0.46;0.82)	0.57	NA	NA	very serious	serious concern	NA	not serious	NA	0	0	Low

Author	Intervention	Control	Outcomes	N RCT	Metric	Effect size (95% CI)	p_value	I²	P_egger	Downgrade factors					Upgrade factors		Quality
										Risk of bias	Imprecision	Inconsistency	Indirectness	Publication bias	SMD >0.8 or <–0.8	SMD >1.2 or <–1.2	
Kang et al. 2022[57]	HNS	Inactive	SBP	1	SMD	–0.56 (–1.17;0.04)	0.07	NA	NA	not serious	serious concern	NA	not serious	NA	0	0	Mod.
Kang et al. 2022[57]	HNS	Inactive	DBP	1	SMD	–0.5 (–1.10;0.11)	0.1	NA	NA	not serious	serious concern	NA	not serious	NA	0	0	Mod.
Alrubasy et al. 2024[59]	HNS	Inactive	AHI	3	SMD	–0.74 (–1.12;0-.37)	<0.0001	50.69	NA	Not serious	Serious concern	NA	Serious concern	NA	0	0	Mod.
Alrubasy et al. 2024[59]	HNS	Inactive	ESS	3	SMD	–0.82 (–1.08;-0.57)	<0.0001	0	NA	Not serious	Serious concern	NA	Serious concern	NA	1	0	Mod.
Alrubasy et al. 2024[59]	HNS	Inactive	QoL	2	SMD	0.57 (0.21;0.93)	0.002	19.15	NA	Not serious	Serious concern	NA	Serious concern	NA	0	0	Mod.
Pépin et al. 2024[80]	Modafinil	Inactive	ESS	12	SMD	–0.69 (–0.98;–0.39)	<0.0001	83.45	0.13	not serious	not serious	serious concern	not serious	not serious	0	0	Mod.
Gao et al. 2025[8]	MAD	positional therapy	AHI	5	SMD	–0.16 (–0.43; 0.11)	0.35	0	NA	very serious	not serious	not serious	serious concern	NA	0	0	Low.
Mohamed et al. 2024[11]	MAD	positional therapy	ESS	3	SMD	0.43 (0.17;0.69)	0.25	47.03	NA	serious concerns	not serious	not serious	not serious	NA	0	0	Mod.
Mohamed et al. 2024[11]	MAD	positional therapy	QoL	3	SMD	0.08 (–0.18;0.33)	0	0	NA	serious concerns	not serious	not serious	not serious	NA	0	0	Mod.
Mohamed et al. 2024[11]	MAD	positional therapy	Adherence	2	SMD	0.13 (–0.14. 0.41)	0.55	0	NA	serious concerns	not serious	not serious	not serious	NA	0	0	Mod.
Gao et al. 2025[8]	MAD	positional therapy	safety (any TEAE)	2	SMD	0.33 (0.03;0.64)	0.03	0	NA	very serious	not serious	not serious	serious concern	NA	0	0	Low.
Pépin et al. 2024[80]	Solriamfetol	Inactive	ESS	2	SMD	–0.88 (–1.09;–0.66)	<0.0001	0	NA	not serious	not serious	not serious	not serious	NA	1	0	high
Pépin et al. 2024[80]	Modafinil	Inactive	QoL	9	SMD	0.49 (0.33;0.66)	<0.0001	54.99	NA	not serious	not serious	not serious	not serious	NA	0	0	high
Pépin et al. 2024[80]	Modafinil	Inactive	Safety (any TEAE)	7	SMD	0.44 (0.15;0.72)	0	72.24	NA	not serious	not serious	not serious	not serious	NA	0	0	high
Pépin et al. 2024[80]	Modafinil	Inactive	Safety (serious TEAE)	5	SMD	–0.05 (–0.81;0.71)	0.9	0	NA	not serious	serious concern	not serious	not serious	NA	0	0	Mod.
Pépin et al. 2024[80]	Solriamfetol	Inactive	QoL	3	SMD	0.5 (0.27;0.72)	<0.0001	31.1	NA	not serious	not serious	not serious	not serious	NA	0	0	Mod.

Author	Intervention	Control	Outcomes	N RCT	Metric	Effect size (95% CI)	p_value	I²	P_egger	Downgrade factors					Upgrade factors		Quality
										Risk of bias	Imprecision	Inconsistency	Indirectness	Publication bias	SMD >0.8 or <–0.8	SMD >1.2 or <–1.2	
Pépin et al. 2024[80]	Solriamfetol	Inactive	Safety (any TEAE)	2	SMD	0.53 (0.32;0.74)	<0.0001	0	NA	not serious	not serious	not serious	not serious	NA	0	0	Mod.
Pépin et al. 2024[80]	Solriamfetol	Inactive	Safety (serious TEAE)	3	SMD	0.00 (–0.86;0.87)	0.99	0	NA	not serious	very serious	not serious	not serious	NA	0	0	Mod.
Pépin et al. 2024[80]	Pitolisant	Inactive	ESS	2	SMD	–0.51 [–0.73;–0.29]				not serious	not serious	not serious	not serious	NA	0	0	high
Pépin et al. 2024[80]	Pitolisant	Inactive	QoL	2	SMD	0.58 (0.38;0.78)	<0.0001	0	NA	not serious	not serious	not serious	not serious	NA	0	0	high
Pépin et al. 2024[80]	Pitolisant	Inactive	Safety (any TEAE)	2	SMD	0.01 (–0.61;0.63)	0.98	70.53	NA	not serious	serious concern	not serious	not serious	NA	0	0	Mod.
Pépin et al. 2024[80]	Pitolisant	Inactive	Safety (serious TEAE)	2	SMD	0.14 (–0.59;0.88)	0.7	0	NA	not serious	serious concern	serious concern	not serious	NA	0	0	Mod.
Yang et al. 2025[66]	Liraglutide	Inactive	AHI	3	SMD	–0.51 [–0.89;–0.12]	0.01	NA	NA	Not serious	not serious	NA	Serious concern	NA	0	0	Mod.
Li et al. 2025[64]	Liraglutide	Inactive	SBP	1	SMD	–0.33 [–0.52;–0.15]	0	NA	NA	Not serious	not serious	NA	Serious concern	NA	0	0	Mod.
Li et al. 2025[64]	Liraglutide	Inactive	DBP	1	SMD	–0.12 [–0.3;0.06]	0.2	NA	NA	Not serious	not serious	NA	Serious concern	NA	0	0	Mod.
Altobaishat et al. 2024	Liraglutide	Inactive	Safety (any TEAE)	1	SMD	0.19 [0.09;0.3]	0	NA	NA	Not serious	not serious	NA	Serious concern	NA	0	0	Mod.
Altobaishat et al. 2024	Liraglutide	Inactive	Safety (serious TEAE)	1	SMD	0.01 [–0.6;0.62]	0.97	NA	NA	Not serious	Serious concern	NA	Serious concern	NA	0	0	Mod.
Li et al. 2025	Tirzepatide	Inactive	AHI	2	SMD	–0.84 [–1;–0.68]	<0.0001	0	NA	Not serious	not serious	NA	Serious concern	NA	1	0	Mod.
Li et al. 2025	Tirzepatide	Inactive	SBP	1	SMD	–0.60 [–0.83;–0.37]		NA	NA	Not serious	not serious	NA	Serious concern	NA	0	0	Mod.

Author	Intervention	Control	Outcomes	N RCT	Metric	Effect size (95% CI)	p_value	I²	P_egger	Downgrade factors					Upgrade factors		Quality
										Risk of bias	Imprecision	Inconsistency	Indirectness	Publication bias	SMD >0.8 or <–0.8	SMD >1.2 or <–1.2	
Li et al. 2025	Tirzepatide	Inactive	DBP	1	SMD	–0.3 [–0.52;–0.07]	0.01	NA	NA	Not serious	not serious	NA	Serious concern	NA	0	0	Mod.
Altobaishat et al. 2024[62]	Tirzepatide	Inactive	Safety (any TEAE)	1	SMD	0.02 [–0.05;0.1]	<0.0001	NA	NA	Not serious	not serious	NA	Serious concern	NA	0	0	Mod.
Altobaishat et al. 2024[62]	Tirzepatide	Inactive	Safety (serious TEAE)	1	SMD	0.17 [–0.36;0.69]	NA	NA	NA	Not serious	Serious concern	NA	Serious concern	NA	0	0	Mod.
Yang et al. 2025[66]	CPAP + Liraglutide	CPAP	AHI	1	SMD	0.5 [–0.46;1.46]	0.28	NA	NA	Not serious	not serious	NA	Serious concern	NA	0	0	Mod.
Li et al. 2025[64]	CPAP + Liraglutide	CPAP	SBP	2	SMD	–0.30 [–0.64;0.03]	0.08	0	NA	Not serious	not serious	NA	Serious concern	NA	0	0	Mod.
Li et al. 2025[64]	CPAP + Liraglutide	CPAP	DBP	2	SMD	–0.24 [–0.58;0.09]	0.15	NA	NA	Not serious	not serious	NA	Serious concern	NA	0	0	Mod.
Li et al. 2025[64]	CPAP + Tirzepatide	CPAP	SBP	3	SMD	–0.26 [–0.48;–0.04]	0.02	NA	NA	Not serious	not serious	NA	Serious concern	NA	0	0	Mod.
Li et al. 2025[64]	CPAP + Tirzepatide	CPAP	DBP	3	SMD	–0.11 [–0.34;–0.10]	0.31	NA	NA	Not serious	not serious	NA	Serious concern	NA	0	0	Mod.
Altobaishat et al. 2024[62]	CPAP + Tirzepatide	CPAP	Safety (any TEAE)	1	SMD	0.07 [0.00;0.15]	0.05	NA	NA	Not serious	not serious	NA	Serious concern	NA	0	0	Mod.
Altobaishat et al. 2024[62]	CPAP + Tirzepatide	CPAP	Safety (serious TEAE)	1	SMD	–0.33 [–0.83;0.17]	0.2	NA	NA	Not serious	Serious concern	NA	Serious concern	NA	0	0	Mod.
Rueda et al. 2020[28]	OMT	CPAP	AHI	1	SMD	0.73 [0.17;1.3]	0.01	NA	NA	Not serious	Serious concern	NA	Serious concern	NA	0	0	Mod.
Rueda et al. 2020[28]	OMT	CPAP	ESS	1	SMD	0.08 [–0.46;0.63]	0.76	NA	NA	Not serious	Serious concern	NA	Serious concern	NA	0	0	Mod.

Author	Intervention	Control	Outcomes	N RCT	Metric	Effect size (95% CI)	p_value	I²	P_egger	Downgrade factors					Upgrade factors		Quality
										Risk of bias	Imprecision	Inconsistency	Indirectness	Publication bias	SMD >0.8 or <–0.8	SMD >1.2 or <–1.2	
Tang et al. 2024[19]	OMT	Inactive	AHI	11	SMD	–0.28 [–0.8;0.25]	0.3	79.4	0.72	not serious	serious concern	not serious	serious concern	NA	0	0	Mod.
Silva de Sousa et al. 2024[27]	OMT	Inactive	DBP	4	SMD	–0.52 [–1.22;0.18]	0.01	39.38	NA	Not serious	Serious concern	not serious	serious concern	NA	0	0	Mod.
Silva de Sousa et al. 2024[27]	OMT	Inactive	SBP	4	SMD	–0.72 [–1.28;0.16]	0.14	65.13	NA	Not serious	Serious concern	not serious	serious concern	NA	0	0	Mod.
Tang et al. 2024[19]	OMT	Inactive	ESS	9	SMD	–0.78 [–1.1; –0.5]	<0.0001	40.86	NA	Not serious	serious concern	not serious	serious concern	NA	0	0	Mod.
Ferreira et al. 2025[87]	OMT + CPAP	CPAP	AHI	2	SMD	–0.13 [–0.56;0.31]	0.57	0	NA	Not serious	Serious concern	not serious	serious concern	NA	0	0	Mod.
Ferreira et al. 2025[87]	OMT + CPAP	CPAP	ESS	2	SMD	0.02 [–0.41;0.45]	0.92	0	NA	Not serious	Serious concern	not serious	serious concern	NA	0	0	Mod.
Ferreira et al. 2025[87]	OMT + PA	Inactive	AHI	1	SMD	–0.38 [–1.18;0.42]	0.34	NA	NA	Not serious	Serious concern	NA	serious concern	NA	0	0	Mod.
Ferreira et al. 2025[87]	OMT + PA	Inactive	ESS	1	SMD	–0.13 [–0.93;0.66]	0.73	NA	NA	Not serious	Serious concern	NA	serious concern	NA	0	0	Mod.

AHI = apnoea-hypopnea index; BP = blood pressure; CPAP = continuous positive airway pressure; CV = cardiovascular; DBP = diastolic blood pressure; ESS = Epworth Sleepiness Scale; HNS = hypoglossal nerve stimulation; MAD = mandibular advancement device; Mod. = moderate; NA = not available; QoL = quality of life; SBP = systolic blood pressure; SMD = standardised mean difference; TEAE = treatment-emergent adverse event; WL = weight loss.

References

1. Basile C, Villaschi A, Maggioni AP. When a meta-analysis can be really useful? *Int J Cardiol* 2025; **436**: 133423.
2. Guyatt GH, Oxman AD, Vist GE, et al. GRADE: an emerging consensus on rating quality of evidence and strength of recommendations. *Bmj* 2008; **336**: 924-6.
3. Hozo SP, Djulbegovic B, Hozo I. Estimating the mean and variance from the median, range, and the size of a sample. *BMC Med Res Methodol* 2005; **5**: 13.
4. Wan X, Wang W, Liu J, Tong T. Estimating the sample mean and standard deviation from the sample size, median, range and/or interquartile range. *BMC Med Res Methodol* 2014; **14**: 135.
5. Luo D, Wan X, Liu J, Tong T. Optimally estimating the sample mean from the sample size, median, mid-range, and/or mid-quartile range. *Stat Methods Med Res* 2018; **27**: 1785-805.
6. Higgins J, Thomas J. Cochrane Handbook for Systematic Reviews of Interventions. Version 6.5, 2024. Available from: https://www.cochrane.org/authors/handbooks-and-manuals/handbook/current. Accessed 15 Jul 2025.
7. Murad MH, Wang Z, Chu H, Lin L. When continuous outcomes are measured using different scales: guide for meta-analysis and interpretation. *BMJ* 2019; **364**: k4817.
8. Gao Y, Zhu S, Li W, Lai Y. Comparative efficacy of sleep positional therapy, oral appliance therapy, and CPAP in obstructive sleep apnea: a meta-analysis of mean changes in key outcomes. *Front Med (Lausanne)* 2025; **12**: 1517274.
9. Srijithesh PR, Aghoram R, Goel A, Dhanya J. Positional therapy for obstructive sleep apnoea. *Cochrane Database Syst Rev* 2019; **5**: Cd010990.
10. Gao YN, Wu YC, Lin SY, Chang JZ, Tu YK. Short-term efficacy of minimally invasive treatments for adult obstructive sleep apnea: A systematic review and network meta-analysis of randomized controlled trials. *J Formos Med Assoc* 2019; **118**: 750-65.
11. Mohamed AM, Mohammed OM, Liu S, et al. Oral appliance therapy vs. positional therapy for managing positional obstructive sleep apnea; a systematic review and meta-analysis of randomized control trials. *BMC Oral Health* 2024; **24**: 666.
12. Martínez Revuelta L, Flores-Fraile J, Zubizarreta-Macho Á, Montiel-Company JM, Lobo-Galindo AB, Arrieta Blanco P. Relationship Between Obstructive Sleep Apnea and Sports-Systematic Review and Meta-Analysis. *J Clin Med* 2024; **13**.

13. Lins-Filho O, Porto Aguiar JL, Vieira de Almeida JR, et al. Effect of exercise training on body composition in patients with obstructive sleep apnea: a systematic review and meta-analysis. *Sleep Med* 2021; **87**: 105-13.
14. Mendelson M, Bailly S, Marillier M, et al. Obstructive sleep apnea syndrome, objectively measured physical activity and exercise training interventions: a systematic review and meta-analysis. *Front Neurol* 2018; **9**: 73.
15. Edwards BA, Bristow C, O'Driscoll DM, et al. Assessing the impact of diet, exercise and the combination of the two as a treatment for OSA: A systematic review and meta-analysis. *Respirology* 2019; **24**: 740-51.
16. Peng J, Yuan Y, Zhao Y, Ren H. Effects of exercise on patients with obstructive sleep apnea: a systematic review and meta-analysis. *Int J Environ Res Public Health* 2022; **19**.
17. Lins-Filho OL, Pedrosa RP, Gomes JML, et al. Effect of exercise training on subjective parameters in patients with obstructive sleep apnea: a systematic review and meta-analysis. *Sleep Med* 2020; **69**: 1-7.
18. Franks KH, Rowsthorn E, Nicolazzo J, et al. The treatment of sleep dysfunction to improve cognitive function: A meta-analysis of randomized controlled trials. *Sleep Med* 2023; **101**: 118-26.
19. Tang R, Pan J, Huang Y, Ren X. Efficacy comparison of aerobic exercise, combined exercise, oropharyngeal exercise and respiratory muscle training for obstructive sleep apnea: A systematic review and network meta-analysis. *Sleep Med* 2024; **124**: 582-90.
20. Carneiro-Barrera A, Díaz-Román A, Guillén-Riquelme A, Buela-Casal G. Weight loss and lifestyle interventions for obstructive sleep apnoea in adults: Systematic review and meta-analysis. *Obes Rev* 2019; **20**: 750-62.
21. Kovács DK, Gede N, Szabó L, et al. Weight reduction added to CPAP decreases blood pressure and triglyceride level in OSA: Systematic review and meta-analysis. *Clin Transl Sci* 2022; **15**: 1238-48.
22. Zhang F, Tian Z, Shu Y, et al. Efficiency of oro-facial myofunctional therapy in treating obstructive sleep apnoea: A meta-analysis of observational studies. *J Oral Rehabil* 2022; **49**: 734-45.
23. Lin HY, Su PL, Lin CY, Hung CH. Models of anatomically based oropharyngeal rehabilitation with a multilevel approach for patients with obstructive sleep apnea: a meta-synthesis and meta-analysis. *Sleep Breath* 2020; **24**: 1279-91.

24. Dar JA, Mujaddadi A, Moiz JA. Effects of inspiratory muscle training in patients with obstructive sleep apnoea syndrome: a systematic review and meta-analysis. *Sleep Sci* 2022; **15**: 480-9.
25. Hsu B, Emperumal CP, Grbach VX, Padilla M, Enciso R. Effects of respiratory muscle therapy on obstructive sleep apnea: a systematic review and meta-analysis. *J Clin Sleep Med* 2020; **16**: 785-801.
26. Cavalcante-Leão BL, de Araujo CM, Ravazzi GC, et al. Effects of respiratory training on obstructive sleep apnea: systematic review and meta-analysis. *Sleep Breath* 2022; **26**: 1527-37.
27. Silva de Sousa A, Pereira da Rocha A, Brandão Tavares DR, et al. Respiratory muscle training for obstructive sleep apnea: Systematic review and meta-analysis. *J Sleep Res* 2024; **33**: e13941.
28. Rueda JR, Mugueta-Aguinaga I, Vilaró J, Rueda-Etxebarria M. Myofunctional therapy (oropharyngeal exercises) for obstructive sleep apnoea. *Cochrane Database Syst Rev* 2020; **11**: Cd013449.
29. Li Z, Cai S, Wang J, Chen R. Predictors of the efficacy for daytime sleepiness in patients with obstructive sleep apnea with continual positive airway pressure therapy: a meta-analysis of randomized controlled trials. *Front Neurol* 2022; **13**: 911996.
30. Wang ML, Wang C, Tuo M, et al. Cognitive effects of treating obstructive sleep apnea: a meta-analysis of randomized controlled trials. *J Alzheimers Dis* 2020; **75**: 705-15.
31. Labarca G, Saavedra D, Dreyse J, Jorquera J, Barbe F. Efficacy of CPAP for improvements in sleepiness, cognition, mood, and quality of life in elderly patients with OSA: systematic review and meta-analysis of randomized controlled trials. *Chest* 2020; **158**: 751-64.
32. Li J, Yan W, Yi M, Lin R, Huang Z, Zhang Y. Efficacy of CPAP duration and adherence for cognitive improvement in patients with obstructive sleep apnea: a meta-analysis of randomized controlled trials. *Sleep Breath* 2023; **27**: 973-82.
33. Khan SU, Duran CA, Rahman H, Lekkala M, Saleem MA, Kaluski E. A meta-analysis of continuous positive airway pressure therapy in prevention of cardiovascular events in patients with obstructive sleep apnoea. *Eur Heart J* 2018; **39**: 2291-7.

34. Zhu B, Ma C, Chaiard J, Shi C. Effect of continuous positive airway pressure on glucose metabolism in adults with type 2 diabetes: a systematic review and meta-analysis of randomized controlled trials. *Sleep Breath* 2018; **22**: 287-95.
35. Yan B, Jin Y, Hu Y, Li S. Effects of continuous positive airway pressure on elderly patients with obstructive sleep apnea: a meta-analysis. *Med Sci (Paris)* 2018; **34 Focus issue F1**: 66-73.
36. Patil SP, Ayappa IA, Caples SM, Kimoff RJ, Patel SR, Harrod CG. Treatment of adult obstructive sleep apnea with positive airway pressure: an American Academy of Sleep Medicine clinical practice guideline. *J Clin Sleep Med* 2019; **15**: 335-43.
37. Timkova V, Nagyova I, Reijneveld SA, Tkacova R, van Dijk JP, Bültmann U. Quality of life of obstructive sleep apnoea patients receiving continuous positive airway pressure treatment: A systematic review and meta-analysis. *Heart Lung* 2020; **49**: 10-24.
38. Zheng D, Xu Y, You S, et al. Effects of continuous positive airway pressure on depression and anxiety symptoms in patients with obstructive sleep apnoea: results from the sleep apnoea cardiovascular Endpoint randomised trial and meta-analysis. *EClinicalMedicine* 2019; **11**: 89-96.
39. Brill AK, Horvath T, Seiler A, et al. CPAP as treatment of sleep apnea after stroke: A meta-analysis of randomized trials. *Neurology* 2018; **90**: e1222-e30.
40. Lv M, Mao J, Wang S, et al. Effect of continuous positive airway pressure on cardiometabolic risk factors in patients with obstructive sleep apnea: A systematic review and meta-analysis. *Respir Med* 2024; **235**: 107852.
41. Shang W, Zhang Y, Wang G, Han D. Benefits of continuous positive airway pressure on glycaemic control and insulin resistance in patients with type 2 diabetes and obstructive sleep apnoea: A meta-analysis. *Diabetes Obes Metab* 2021; **23**: 540-8.
42. Shang W, Zhang Y, Liu L, Chen F, Wang G, Han D. Benefits of continuous positive airway pressure on blood pressure in patients with hypertension and obstructive sleep apnea: a meta-analysis. *Hypertens Res* 2022; **45**: 1802-13.
43. Liu J, Xu J, Guan S, Wang W. Effects of different treatments on metabolic syndrome in patients with obstructive sleep apnea: a meta-analysis. *Front Med (Lausanne)* 2024; **11**: 1354489.

44. Labarca G, Dreyse J, Salas C, Letelier F, Jorquera J. A validation study of four different cluster analyses of OSA and the incidence of cardiovascular mortality in a Hispanic population. *Chest* 2021; **160**: 2266-74.
45. Lei Q, Lv Y, Li K, et al. Effects of continuous positive airway pressure on blood pressure in patients with resistant hypertension and obstructive sleep apnea: a systematic review and meta-analysis of six randomized controlled trials. *J Bras Pneumol* 2017; **43**: 373-9.
46. Feng J, Li K, Luo W, Xie F, Li M, Wu Y. Effect of continuous positive pressure ventilation on left ventricular diastolic function E/A ratio in patients with obstructive sleep apnea: a meta-analysis. *Sleep Breath* 2023; **27**: 2333-40.
47. Sun L, Chang YF, Wang YF, et al. Effect of continuous positive airway pressure on blood pressure in patients with resistant hypertension and obstructive sleep apnea: an updated meta-analysis. *Curr Hypertens Rep* 2024; **26**: 201-11.
48. Li P, Ning XH, Lin H, Zhang N, Gao YF, Ping F. Continuous positive airway pressure versus mandibular advancement device in the treatment of obstructive sleep apnea: a systematic review and meta-analysis. *Sleep Med* 2020; **72**: 5-11.
49. Schwartz M, Acosta L, Hung YL, Padilla M, Enciso R. Effects of CPAP and mandibular advancement device treatment in obstructive sleep apnea patients: a systematic review and meta-analysis. *Sleep Breath* 2018; **22**: 555-68.
50. Kuhn E, Schwarz EI, Bratton DJ, Rossi VA, Kohler M. Effects of CPAP and mandibular advancement devices on health-related quality of life in OSA: a systematic review and meta-analysis. *Chest* 2017; **151**: 786-94.
51. Yu M, Ma Y, Han F, Gao X. Long-term efficacy of mandibular advancement devices in the treatment of adult obstructive sleep apnea: A systematic review and meta-analysis. *PLoS One* 2023; **18**: e0292832.
52. Guo MY, Li PJ, Xiao Y, Cao Y, Liang ZA. Effectiveness of mandibular advancement devices in the treatment of obstructive sleep apnea and the impact of different body positions on treatment： A systematic review and meta-analysis. *Sleep Med* 2024; **113**: 275-83.
53. Vila-Nova TEL, de Luna Gomes JM, do Egito Vasconcelos BC, Pellizzer EP, Moraes SLD. The influence of nocturnal use of complete dentures on cardiorespiratory parameters

of patients with obstructive sleep apnea: a systematic review and meta-analysis. *Clin Oral Investig* 2022; **26**: 4675-86.

54. Rangarajan H, Padmanabhan S, Ranganathan S, Kailasam V. Impact of oral appliance therapy on quality of life (QoL) in patients with obstructive sleep apnea - a systematic review and meta-analysis. *Sleep Breath* 2022; **26**: 983-96.
55. Vimal J, Dutt P, Singh N, Singh BP, Chand P, Jurel S. To compare different non-surgical treatment modalities on treatment of obstructive sleep apnea: A systematic review and meta-analysis. *J Indian Prosthodont Soc* 2022; **22**: 314-27.
56. Belanche Monterde A, Zubizarreta-Macho Á, Lobo Galindo AB, Albaladejo Martínez A, Montiel-Company JM. Mandibular advancement devices decrease systolic pressure during the day and night in patients with obstructive sleep apnea: A systematic review and meta-analysis. *Sleep Breath* 2024; **28**: 1037-49.
57. Kang KT, Yeh TH, Ko JY, Lee CH, Lin MT, Hsu WC. Effect of sleep surgery on blood pressure in adults with obstructive sleep apnea: A Systematic Review and meta-analysis. *Sleep Med Rev* 2022; **62**: 101590.
58. Wollny M, Heiser C, Sommer U, Schöbel C, Braun M. Adverse Events with Hypoglossal Nerve Stimulation in the Treatment of Obstructive Sleep Apnea-A Systematic Review of Clinical Trials and Real-World Data. *J Clin Med* 2024; **13**.
59. Alrubasy WA, Abuawwad MT, Taha MJJ, et al. Hypoglossal nerve stimulation for obstructive sleep apnea in adults: An updated systematic review and meta-analysis. *Respir Med* 2024; **234**: 107826.
60. Ratneswaran D, Guni A, Pengo MF, et al. Electrical stimulation as a therapeutic approach in obstructive sleep apnea - a meta-analysis. *Sleep Breath* 2021; **25**: 207-18.
61. Braun M, Stoerzel M, Wollny M, Schoebel C, Ulrich Sommer J, Heiser C. Patient-reported outcomes with hypoglossal nerve stimulation for treatment of obstructive sleep apnea: a systematic review and meta-analysis. *Eur Arch Otorhinolaryngol* 2023; **280**: 4627-39.
62. Altobaishat O, Farid Gadelmawla A, Balbaa E, Turkmani M, Abouzid M. Safety and efficacy of glucagon-like peptide-1 receptor agonists in patients with obstructive sleep apnea: a systematic review and meta-analysis of randomized controlled trials. *Eur Clin Respir J* 2025; **12**: 2484048.

63. Kow CS, Ramachandram DS, Hasan SS, Thiruchelvam K. Efficacy and safety of GLP-1 receptor agonists in the management of obstructive sleep apnea in individuals without diabetes: A systematic review and meta-analysis of randomized, placebo-controlled trials. *Sleep Med* 2025; **129**: 40-4.
64. Li M, Lin H, Yang Q, et al. Glucagon-like peptide-1 receptor agonists for the treatment of obstructive sleep apnea: a meta-analysis. *Sleep* 2025; **48**.
65. Dutta D, Jindal R, Raizada N, Nagendra L, Kamrul HA, Sharma M. Efficacy and Safety of Glucagon Like Peptide-1 Receptor Agonism Based Therapies in Obstructive Sleep Apnoea: A Systematic Review and Meta-Analysis. *Indian J Endocrinol Metab* 2025; **29**: 4-12.
66. Yang R, Zhang L, Guo J, et al. Glucagon-like Peptide-1 receptor agonists for obstructive sleep apnea in patients with obesity and type 2 diabetes mellitus: a systematic review and meta-analysis. *J Transl Med* 2025; **23**: 389.
67. Bardóczi A, Matics ZZ, Turan C, et al. Efficacy of incretin-based therapies in obesity-related obstructive sleep apnea: a systematic review and meta-analysis of randomized controlled trials. *Sleep Med Rev* 2025; **82**: 102119.
68. John CR, Gandhi S, Sakharia AR, James TT. Maxillomandibular advancement is a successful treatment for obstructive sleep apnoea: a systematic review and meta-analysis. *Int J Oral Maxillofac Surg* 2018; **47**: 1561-71.
69. Camacho M, Nesbitt NB, Lambert E, et al. Laser-assisted uvulopalatoplasty for obstructive sleep apnea: a systematic review and meta-analysis. *Sleep* 2017; **40**.
70. Lechien JR, Chiesa-Estomba CM, Fakhry N, et al. Surgical, clinical, and functional outcomes of transoral robotic surgery used in sleep surgery for obstructive sleep apnea syndrome: A systematic review and meta-analysis. *Head Neck* 2021; **43**: 2216-39.
71. Saenwandee P, Neruntarat C, Saengthong P, et al. Barbed pharyngoplasty for obstructive sleep apnea: A meta-analysis. *Am J Otolaryngol* 2022; **43**: 103306.
72. Schoustra E, van Maanen P, den Haan C, Ravesloot MJL, de Vries N. The role of isolated nasal surgery in obstructive sleep apnea therapy-a systematic review. *Brain Sci* 2022; **12**.

73. Zhou N, Ho JTF, Huang Z, et al. Maxillomandibular advancement versus multilevel surgery for treatment of obstructive sleep apnea: A systematic review and meta-analysis. *Sleep Med Rev* 2021; **57**: 101471.
74. He M, Yin G, Zhan S, et al. Long-term efficacy of uvulopalatopharyngoplasty among adult patients with obstructive sleep apnea: a systematic review and meta-analysis. *Otolaryngol Head Neck Surg* 2019; **161**: 401-11.
75. Zhang Y, Wang W, Yang C, Shen J, Shi M, Wang B. Improvement in nocturnal hypoxemia in obese patients with obstructive sleep apnea after bariatric surgery: a meta-analysis. *Obes Surg* 2019; **29**: 601-8.
76. Locke BW, Gomez-Lumbreras A, Tan CJ, et al. The association of weight loss from anti-obesity medications or bariatric surgery and apnea-hypopnea index in obstructive sleep apnea. *Obes Rev* 2024; **25**: e13697.
77. Kou C, Zhao X, Lin X, Fan X, Wang Q, Yu J. Effect of different treatments for obstructive sleep apnoea on blood pressure. *J Hypertens* 2022; **40**: 1071-84.
78. Wong AM, Barnes HN, Joosten SA, et al. The effect of surgical weight loss on obstructive sleep apnoea: A systematic review and meta-analysis. *Sleep Med Rev* 2018; **42**: 85-99.
79. Malhotra A, Grunstein RR, Fietze I, et al. Tirzepatide for the treatment of obstructive sleep apnea and obesity. *N Engl J Med* 2024; **391**: 1193-205.
80. Pépin J-L, Lehert P, Ben Messaoud R, et al. Comparative efficacy, safety and benefit/risk of alerting agents for excessive daytime sleepiness in patients with obstructive sleep apnoea: a network meta-analysis. *eClinicalMedicine* 2024; **76**: 102843.
81. Wang J, Li X, Yang S, et al. Pitolisant versus placebo for excessive daytime sleepiness in narcolepsy and obstructive sleep apnea: A meta-analysis from randomized controlled trials. *Pharmacol Res* 2021; **167**: 105522.
82. Pitre T, Mah J, Roberts S, et al. Comparative efficacy and safety of wakefulness-promoting agents for excessive daytime sleepiness in patients with obstructive sleep apnea : a systematic review and network meta-analysis. *Ann Intern Med* 2023; **176**: 676-84.

83. Wang J, Yang S, Li X, et al. Efficacy and safety of solriamfetol for excessive sleepiness in narcolepsy and obstructive sleep apnea: findings from randomized controlled trials. *Sleep Med* 2021; **79**: 40-7.
84. Ronnebaum S, Bron M, Patel D, et al. Indirect treatment comparison of solriamfetol, modafinil, and armodafinil for excessive daytime sleepiness in obstructive sleep apnea. *J Clin Sleep Med* 2021; **17**: 2543-55.
85. Subedi R, Singh R, Thakur RK, K CB, Jha D, Ray BK. Efficacy and safety of solriamfetol for excessive daytime sleepiness in narcolepsy and obstructive sleep apnea: a systematic review and meta-analysis of clinical trials. *Sleep Med* 2020; **75**: 510-21.
86. Neshat SS, Heidari A, Henriquez-Beltran M, et al. Evaluating pharmacological treatments for excessive daytime sleepiness in obstructive sleep apnea: A comprehensive network meta-analysis and systematic review. *Sleep Med Rev* 2024; **76**: 101934.
87. Ferreira LGDA, Miranda VSG, Baseggio MEP, Marcolino MAZ, Vidor DCGM. Myofunctional Therapy for the Treatment of Obstructive Sleep Apnea: Systematic Review and Meta-Analysis. *Int Arch Otorhinolaryngol* 2025; **29**: 1-10.
88. Zhang M, Liu Y, Liu Y, et al. Effectiveness of oral appliances versus continuous positive airway pressure in treatment of OSA patients: An updated meta-analysis. *Cranio* 2019; **37**: 347-64.
89. de Vries GE, Wijkstra PJ, Houwerzijl EJ, Kerstjens HAM, Hoekema A. Cardiovascular effects of oral appliance therapy in obstructive sleep apnea: A systematic review and meta-analysis. *Sleep Med Rev* 2018; **40**: 55-68.
90. Sterne JAC, Savović J, Page MJ, et al. RoB 2: a revised tool for assessing risk of bias in randomised trials. *BMJ* 2019; **366**: l4898.
